# Inhibition of DNA Topoisomerase Type II*α* (TOP2A) by Mitoxantrone and Its Halogenated Derivatives: A Combined Density Functional and Molecular Docking Study

**DOI:** 10.1155/2016/6817502

**Published:** 2016-02-15

**Authors:** Md. Abu Saleh, Md. Solayman, Mohammad Mazharol Hoque, Mohammad A. K. Khan, Mohammed G. Sarwar, Mohammad A. Halim

**Affiliations:** ^1^Bangladesh Institute of Computational Chemistry and Biochemistry, 38 Green Road West, Dhaka 1205, Bangladesh; ^2^Department of Biochemistry and Molecular Biology, Jahangirnagar University, Dhaka 1342, Bangladesh; ^3^Jubail University College, Department of General Studies, Jubail 31961, Saudi Arabia; ^4^Department of Chemistry, The Scripps Research Institute, 10550 North Torrey Pines Road, MB26, La Jolla, CA 92037, USA; ^5^Institut Lumière Matière, Université Lyon 1-CNRS, Université de Lyon, 69622 Villeurbanne Cedex, France

## Abstract

In this study, mitoxantrone and its halogenated derivatives have been designed by density functional theory (DFT) to explore their structural and thermodynamical properties. The performance of these drugs was also evaluated to inhibit DNA topoisomerase type II*α* (TOP2A) by molecular docking calculation. Noncovalent interactions play significant role in improving the performance of halogenated drugs. The combined quantum and molecular mechanics calculations revealed that CF_3_ containing drug shows better preference in inhibiting the TOP2A compared to other modified drugs.

## 1. Introduction

Cancer is one of the most devastating diseases and causes of million deaths, and it is predicted to continue to be catastrophic in the coming years [1]. Surgery and radiation therapies are limited to treating cancers that are confined to highly precise areas. Chemotherapy is advantageous over such treatments because of its ability to treat widespread or metastatic cancers. Chemotherapy, a key way to treat the malignant tumors, uses various chemical agents to destroy cancer cells [2]. The chemotherapy treatment has earned ample attention and a great deal of recent efforts have been concentrating on the design and development of varied anticancer drugs. Mitoxantrone (1,4-dihydroxy-5,8-bis({2-[(2-hydroxyethyl)amino]ethyl}amino)-9,10-dihydroanthracene-9,10-dione) is a potent synthetic anticancer drug which blocks DNA synthesis by inhibiting the function of DNA topoisomerase II [3–5]. The drug was selected amongst a series of anthracenedione derivatives that have structural similarities to the anthracyclines. Due to the absence of amino-sugar moiety in mitoxantrone, it has less cardio-toxicity [6]. In several cardio-toxicity models, mitoxantrone appeared to have less toxicity than doxorubicin [7]. Currently, mitoxantrone has been used for treating different type of cancers including breast cancer, leukemia, lymphoma, and prostate cancer [8]. Moreover, this drug provides a new therapeutic option for patients with worsening relapsing-remitting and secondary progressive multiple sclerosis and hepatocellular carcinoma [9, 10].

The widely used target of existing anticancer drugs including mitoxantrone is DNA topoisomerase type II*α* (TOP2A) and the expression of this enzyme has been used as cancer cell marker because of its role in cell proliferation [11–13]. During DNA replication, TOP2A plays a key role and its main functions are chromosome segregation and chromosome condensation [14]. Humans express another isoform of topoisomerase II which is known as DNA topoisomerase II *β* (TOP2B) [15, 16]. The two isoforms of topoisomerase II are 68% [17] identical and their catalytic portion share ~78% similarity. ATP dependent type II topoisomerases [18–21] operate by a complex mechanism that involves the organized association and dissociation of subunit dimerization elements [22–25]. For this cleavage reaction, one segment of the DNA duplex (defined as “G-segment”) is bound and cleaved by the enzyme whereas a second double stranded DNA (defined as “T-segment”) is transported through the break. The assembly of topoisomerase and DNA is called the cleavage complex in which a pair of symmetrically related tyrosine residues (Tyr) is responsible for G-segment breakage [26, 27]. Eukaryotic TOP2A contains three regions known as the N-gate, DNA-gate, the C-gate, and the catalytic Tyr805, which is responsible for cleavage present in the DNA-gate [28, 29]. The clinically active anticancer agent, mitoxantrone, inhibits topoisomerase II by increasing its levels in TOP2-DNA complexes [30, 31]. In addition, antitopoisomerase agents that bind within the DNA-gate either impede or stabilize the cleavage and relegation events [32].

Nonbonding interactions between the drug and amino acid residues of the receptor play a crucial role in preventing/obstructing the active/enzymatic site(s), which are responsible for causing certain diseases. Various nonbonding interactions have been identified in drug-receptor complex including hydrogen bonding, halogen bonding, cation-*π* interactions, anion-pi interactions, pi-alkyl interaction, *π*-*π* stacking, and T-shape interactions [33–36]. Molecular level interpretation of these nonbonding interactions appeared as a key factor to design superior drug which can effectively inhibit the receptor protein.

In this study, quantum mechanical calculations were carried out to model and explore the structural, thermodynamical, and molecular orbital properties of 10 halogenated mitoxantrone drugs. Moreover, the binding affinity and nonbonding interactions of these drugs with TOP2A are evaluated by molecular docking study.

## 2. Computational Methods

### 2.1. Drug Design by Quantum Mechanical Calculations

All electronic calculations were carried out using Gaussian 09 program package [37]. The initial geometry of 3D structure of mitoxantrone (D) was taken from PubChem Open Chemistry Database [38]. The structure of mitoxantrone was fully optimized by density functional theory employing Becke's (B3) [39, 40] exchange functional combining Lee, Yang, and Parr's (LYP) correlation functional [41]. For all modified drug molecules (D1–D10), Cramer and Truhlar's MidiX basis set was employed [42]. MidiX basis set is originally developed from the Huzinaga MidiX basis and applied to H, C-F, S-Cl, Br, and I atoms. The MidiX basis set is comparatively smaller than the popular 6–31G(d,p) and can provide excellent geometries and charge balances with reasonable computational time and accuracy [43].

After optimization, subsequent vibrational frequency calculation was performed in order to confirm that the stationary points correspond to minima on the potential energy surface. Electronic energies, enthalpies, Gibb's free energies, dipole moments, and partial charge analysis were also explored for all optimized-energy geometries. Molecular orbital calculations were performed at the same level of theory. Hardness and softness of all drugs were also determined from the energies of frontier HOMOs and LUMOs. Considering Parr and Pearson interpretation [43–45] of DFT and Koopmans theorem [46] on the correlation of ionization potential (*I*) and electron affinities (*E*) with HOMO and LUMO energies (*ε*), hardness (*η*) and softness (*S*) of the drugs were calculated according to the following equation:

(1) η=εLUMO−εHOMO2,S=1η.

### 2.2. Preparation of Protein

The mitoxantrone and all modified drugs were subjected to molecular docking against human topoisomerase II*α* (TOP2A). The crystal structure of TOP2A was collected from the Protein Data Bank (PDB) database (PDB ID: 4FM9; Chain A) [32]. Since the crystal structure has some issues related to improper bond order, side chains geometry, and missing hydrogen atoms, the structure was checked and an energy minimization was performed with the Swiss-Pdb Viewer software packages (version 4.1.0) [47]. Prior to docking, all the heteroatoms and water molecules were removed from the crystal structure using PyMol (version 1.3) software packages [48]. Addition of nonpolar hydrogen atoms is performed by AutoDock Tools (ADT) of MGL software packages (version 1.5.6). Subsequently the fully optimized structures of the halogenated compounds were opened using ADT to add Gasteiger charges and to set TORSDOF followed by the conversion of all rotatable bonds into nonrotatable (rigid). Finally, both the proteins and ligand structures were saved in  .pdbqt format as it is the only one supported file format that required by AutoDock Vina software (version 1.1.2, May 11, 2011) for docking analysis [49].

### 2.3. Binding Site and Docking

The active binding pocket of TOP2A is predicted by CastP [50] having the highest pocket area and volume that are 4390 Å^2^ and 8674.2 Å^3^, respectively. The binding pocket and the amino acid residues are presented in Figure S1 (supporting information; see Supplementary Material available online at http://dx.doi.org/10.1155/2016/6817502). The binding site residues predicted by CastP for TOP2A were used for the generation of the grid box.

To dock the mitoxantrone and its halogenated derivatives against TOP2A, the center of the grid box was set at 33.5565, 41.4725, and 15.9145 Å and the box size was set at 25, 25, and 25 Å in *x*, *y*, and *z* directions, respectively. Autodock Vina docking protocol was employed to conduct the docking study. Next, the docked pose of lowest binding free energy conformer with the respective protein was analyzed using PyMOL Molecular Graphics System (version 1.3) [48], Accelrys Discovery Studio 4.1 [51], and LigPlot+ version v1.4.5 [52].

## 3. Results and Discussions

**Figure 1:**
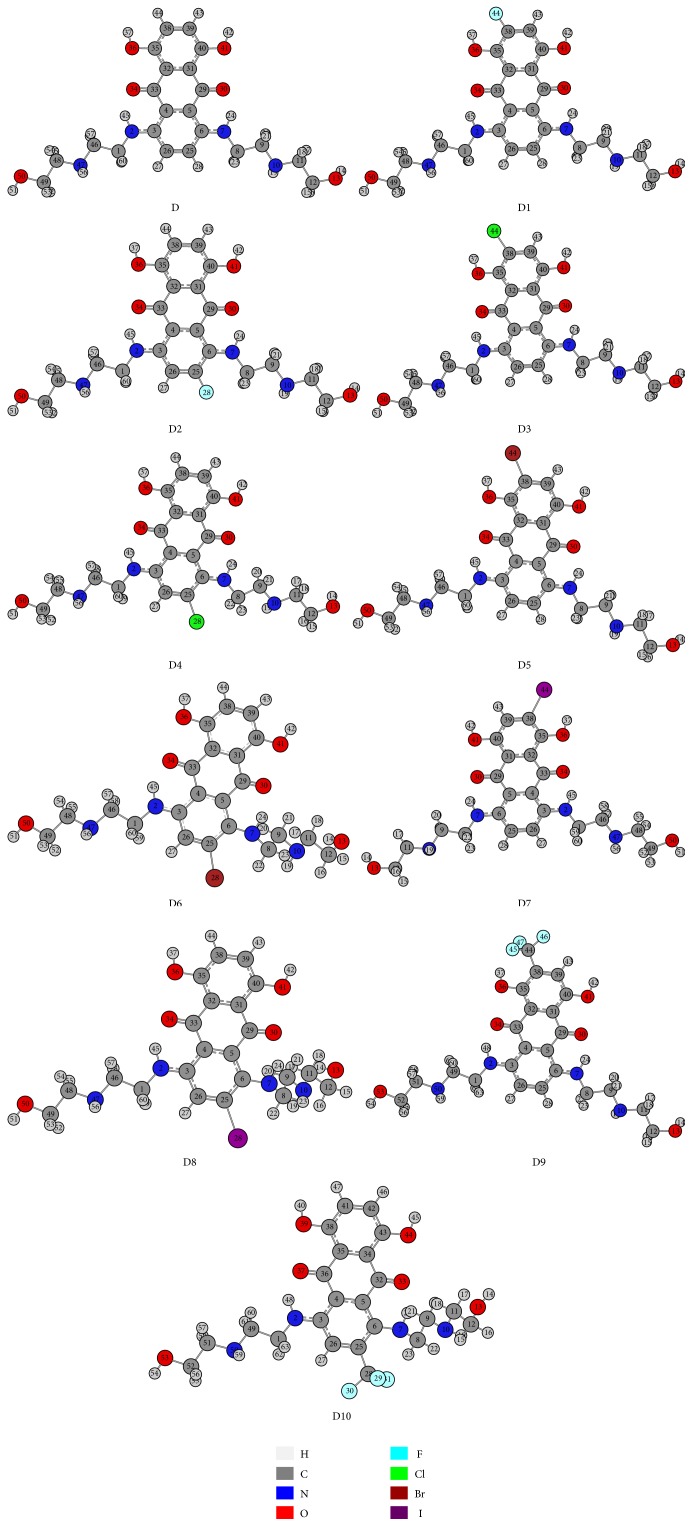
Optimized structure of mitoxantrone (D) and its halogenated derivatives (D1, D2, D3, D4, D5, D6, D7, D8, D9, and D10) calculated at B3LYP/MidiX level of theory.

**Figure 2:**
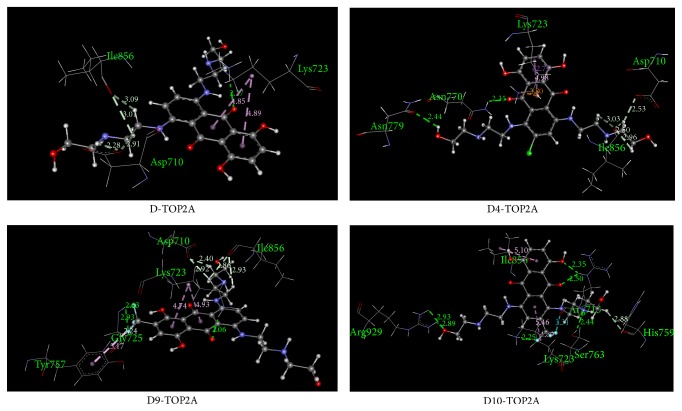
Nonbonding interaction of D, D4, D9, and D10 with TOP2A.

The optimized structures of mitoxantrone (D) and its halogenated derivatives (D1–D10) computed at the B3LYP/MidiX level of theory are presented in Figure 1. Partial charges and direction of the dipole moments of all drugs are illustrated in the supplementary Figure S2. The stoichiometry, electronic energy, enthalpy, Gibbs free energy, and dipole moment of all drugs are reported in Table 1. The HOMO and LUMO energies, HOMO-LUMO gap, hardness, and softness of all drugs are summarized in Table 2. The pictographical presentation of all frontier orbitals is displayed in supplementary Figure S3. The binding affinity and all nonbonding interactions of all drug-receptor complexes are summarized in Table 3 and Figure 2. Aromatic and hydrophobic surface of binding pocket are shown in supplementary information (Figures S4 and S5).

### 3.1. The Electronic Structure of Mitoxantrone and Its Halogenated Derivatives

Installation of F, Cl, Br, I, and CF_3_ on mitoxantrone at positions 44 and 28 significantly influences the structural properties of these drugs in terms of energy, partial charge distribution, and dipole moment. The electronic energy, enthalpy, and Gibbs free energy appear to be more negative following halogenation which indicates that structures become more stable after modification (Table 1). The highest Gibbs free energy is observed for D5. In D5, bromination at position 44, replacing the hydrogen atom, changes the free energy to −4077.7124 Hartree from −1516.2828 Hartree. The highest electronic energy and enthalpy are also detected for D5.

In general, the polar nature of the molecule provides a higher value of the dipole moment. This parameter is a good indicator for studying the drug-receptor interaction [53] and plays a significant role on the formation of hydrogen bonds in biological systems. Incorporation of F, Br, I, and CF_3_ at position 44, replacing the hydrogen atom, decreases the dipole moment. To the contrary, increased values of the dipole moment are observed when F, Br, I, and CF_3_ groups are installed at position 25 (Table 1). Moreover, Table 1 shows that the dipole moment of D9 is 1.6814 Debye which is closer to that of mitoxantrone (1.5477 Debye).

The two global chemical descriptors known as hardness and softness are also calculated for all drugs and presented in Table 2. It is observed that D9 has the highest softness and lowest hardness in comparison to mitoxantrone. Among all the modified drugs, the lowest HOMO-LUMO gap has been observed for D9. The lowest HOMO-LUMO gap indicates that the molecule is more chemically reactive [54]. Pearson found that the HOMO-LUMO gap has a relation to the chemical hardness and softness of a molecule [55, 56].

The partial charges on the halogen atom of the modified drugs are changed due to their position. In modified drugs D1, D3, D5, and D7 the partial charges of F, Cl, Br, and I in the 44 position are −0.324 (a.u), −0.218 (a.u), −0.066 (a.u), and +0.125 (a.u), respectively. It is interesting to note that iodine bears partial positive charge. On the other hand, in D2, D4, D6, and D8 drugs, the partial charges are changed to −0.319 (a.u), −0.208 (a.u), −0.048 (a.u), and +0.133 (a.u), respectively. Several recent studies showed that halogen (particularly Br and I) atoms can form a halogen bond similar to a hydrogen bond and these noncovalent interactions can play remarkable roles in biological and chemical systems [57–62]. In halogen bonding, the X atom can act as an electron deficient Lewis acid and this acid is attracted by Lewis bases that are electron rich (such as the carbonyl oxygen and amine nitrogen). In our study, we notice that the I atom of modified drugs D7 and D8 shows positive charge of +0.125 (a.u) and +0.133 (a.u), respectively.

### 3.2. Interaction and Binding Affinity of Mitoxantrone (D) and Modified Drug (D4) against TOP2A

**Figure 3:**
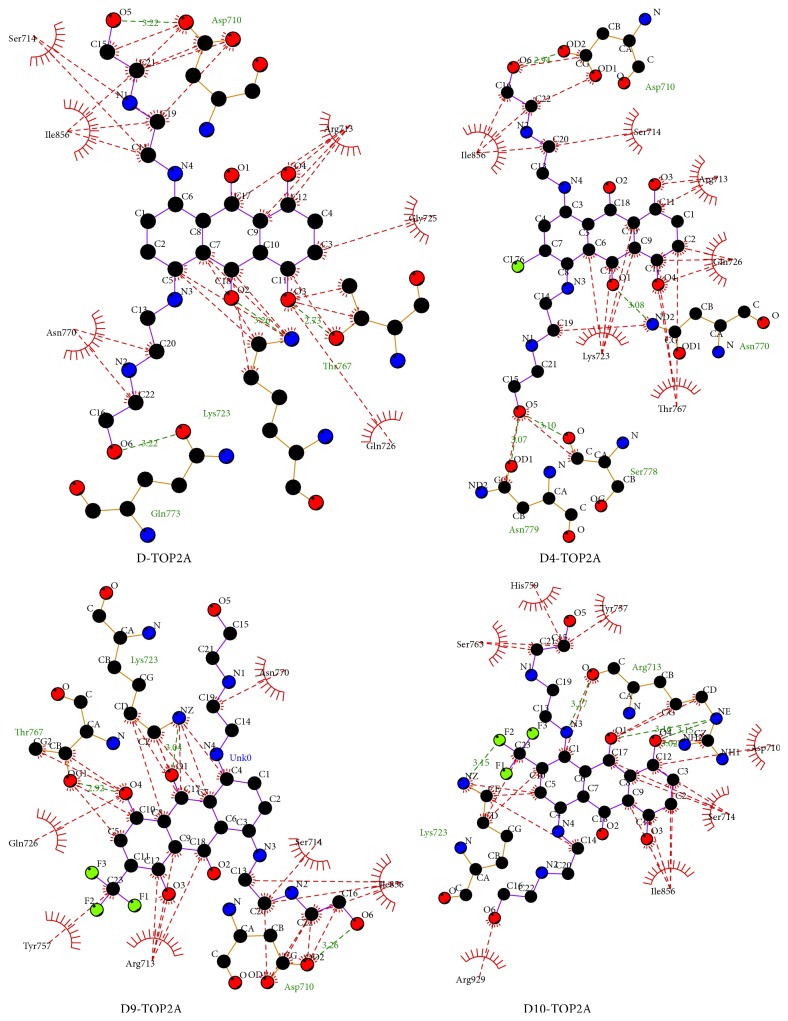
D, D4, D9, and D10 interactions with surrounding residues of TOP2A generated by LigPlus.

The binding affinity of D and D4 against TOP2A is −9.2 and −10.3 kcal mol^−1^, respectively. The surrounding residues (generated by LigPlot program) of TOP2A which interact with D and D4 are demonstrated in Figure 3. Both drugs have significant interaction with amino acid residues such as Ile, Lys, and Gly. Details of nonbonding interactions are examined by Discovery Studios Software. In the D-TOP2A complex, no halogen bond is detected. There are two pi-alkyl interactions (4.89 Å and 4.85 Å) observed between the aromatic ring of D and Lys723. Frontier molecular orbital calculations revealed that the LUMO orbital of D contributes the second pi-alkyl interaction. Both conventional and nonconventional hydrogen bonds are observed in D-TOP2A complex. The C-H⋯O interaction, known as nonconventional hydrogen bond, slightly weaker than its classical O-H⋯O hydrogen bonding, is believed to be critical in a large number of biomacromolecules' crystal structures [63, 64]. Five hydrogen bonds are observed in which four hydrogen bonds are nonconventional (C-H⋯O) and these bonds are formed with Ile856 (3.09 Å), Asp710 (2.91 Å), Ile856 (3.01 Å), and Asp710 (2.28 Å) (Figure 2). This nonconventional hydrogen bond plays crucial role in biological systems.

The D4-TOP2A complex is stabilized by one electrostatic, two hydrophobic, and six hydrogen bonds. Strong pi-cation interaction is detected between N-H of Lys723 (2.80 Å) and benzene ring of D4. The Lys723 residue also participates in the hydrophobic interaction in which one is sigma-pi (2.71 Å) and another one is alkyl-pi (4.95 Å) interaction. In the D4-TOP2A complex, two strong C-H⋯O (distance of 2.96 Å and 2.50 Å) interactions and one relatively weak interaction with a distance of 3.03 Å has been detected with Ile856.

### 3.3. Interaction and Binding Affinity of Modified Drugs D1, D2, D3, D5, D6, D7, and D8 against TOP2A

The binding affinities of D1, D2, and D3 are −9.5, −9.4, and −9.8 kcal mol^−1^, respectively (Table 3). The surrounding residues (generated by LigPlot program) of TOP2A which interact with D1–D3 and D5–D8 are demonstrated in Figure S6. In D1 and D2, incorporation of fluorine at position 44 and 28 by replacing the hydrogen atom does not significantly change the binding affinity. An electrostatic interaction (anion-pi interaction) with a distance of 3.99 Å is found in the D1-TOP2A complex between CO of Glu839 and aromatic ring of D1. In the D1-TOP2A complex, Glu839, Phe1003, Ile715, Glu712, Glu839, His1005, and Val1006 are actively involved in the noncovalent interaction (Figure S7). Pi-pi stacked interaction between aromatic ring of Phe1003 and D1 plays a crucial role in the D1-TOP2A complex.

In the D2-TOP2A complex, only two amino acids (Lys723 and Asp710) participate in noncovalent interactions and no other interactions are detected (Figure S8). In D3-TOP2A complex, two unusual hydrophobic interactions are identified between Cl of D3 and CH2 of Val836 and another is formed by the imidazole ring of His758 with Cl of D3 (Figure S9). Hydrogen bonding plays a significant role on the binding affinity in which Ser709, Ser756, Asp545, His759, Glu839, and Gln544 residues are involved. Electrostatic interactions are also formed by Lys728 and Asp831.

The binding affinities of D5, D6, D7, and D8 against TOP2A are −9.3, −9.9, −9.5, and −9.7 kcal mol^−1^, respectively. These binding energies are slightly higher than that of mitoxantrone. No halogen bond is detected for modified drugs D5-D8 (Figures S10–S13). A strong electrostatic interaction is observed in both modified drugs D6 (2.90 Å) and D8 (2.76 Å) which are formed by Lys723. Pi-alkyl interactions are common for drugs D5, D8, and D9 due to Lys723. In the D5-TOP2A complex, Lys723, Ile856, and Asp710 are responsible for forming hydrogen bonds. However, in the D6-TOP2A complex, Asn770, Asn710, Gly852, and Ile856 residues are involved in hydrogen bonding (Figure S11). Hydrogen boning plays a noticeable role on the binding affinity of D7 and D8 with TOP2A (Figures S12 and S13).

### 3.4. Interaction and Binding Affinity of Modified Drugs D9 and D10 against TOP2A

In modified drugs D9 and D10, the trifluoromethyl group (CF_3_) has been added to carbon (position 38 and 25, resp.) by replacing hydrogen atom (Figure 1). Addition of CF_3_ to organic molecules has wide application in polymers, pharmaceuticals products, material science, and agriculture [65–68]. Due to enhanced binding selectivity, increased lipophilicity, elevated electronegativity, and improved metabolic stability, this group has earned great attention in drug design [69].

The binding affinities of D9 and D10 against TOP2A are −10.3 and −10.0 kcal mol^−1^, respectively (Table 3). The D9-TOP2A complex is stabilized by five hydrogen bonds, three hydrophobic interaction, and three halogen bonds. Three fluorine bonds are formed between fluorine atoms of CF_3_ and Gly725 (Table 3). Moreover, CF_3_ formed one pi-alkyl interaction (5.17 Å) with the aromatic ring of Tyr757. In the D9-TOP2A complex, there is only one conventional hydrogen bond in which the CO group of D9 acts as hydrogen bond acceptor and the NH of Lys723 acts as hydrogen bond donor. Moreover, the HOMO orbital of D1 on CO plays a remarkable role on the formation of this hydrogen bond (Figure S3). The CH_2_ of Lys723 contributes two pi-alkyl interactions (Figure S4) with a distance of 4.74 Å and 4.93 Å. Moreover, Asp710 and Ile856 amino acids form two nonconventional hydrogen bonds (C-O⋯H-C) separately with D9 (Figure 2).

Noncovalent interactions such as hydrogen bonding, halogen bonding, and hydrophobic interactions contributed more towards the binding affinity of TOP2A with modified drug D10. The LUMO region of 38O plays a significant role in the formation of the conventional hydrogen bond (2.50 Å) between the CO of D10 and NH of Arg713. In addition, another hydrogen bond is formed between Arg713 and D10 with a distance of 2.35 Å. In the D10-TOP2A complex, three pi-alkyl interactions are detected in which two are contributed by Lys723 (Table 3). One halogen bond is also observed with a distance of 3.37 Å between CO of Arg713 and F of D10. Among all modified drugs, more conventional hydrogen bonds are found in the D10-TOP2A complex.

## 4. Conclusion

This study reveals that halogenated mitoxantrone drugs also interact with TOP2A. Density functional theory calculation demonstrated some interesting features related to charge distribution, dipole moment, enthalpy, free energy, and molecular orbital of the drug molecules. Halogenation made the modified drugs thermodynamically more stable, evident from the enthalpy and Gibbs free energies. The HOMO-LUMO gap of the modified drugs D3, D5, D7, and D9 is reasonably lower than that of mitoxantrone which indicates that these compounds are more chemically reactive than the unmodified one. The dipole moment of D9 is slightly increased but close to mitoxantrone and the HOMO-LUMO gap is significantly lower than mitoxantrone. Modified drug D9 has the greatest softness amongst all drugs considered. The strongest binding affinity (−10.3 kcal mol^−1^) has been found for D4-TOP2A and D9-TOP2A which is higher than the parent drug. DFT and molecular docking results demonstrated that D4 and D9 show the better performance on inhibiting human topoisomerase 2 alpha. The details of nonbonding interactions detected between the modified drugs and TOP2A may help to develop new anticancer drug which can effectively target the TOP2A receptor.

## Supplementary Material

The binding pocket of the protein; dipole moment, partial charge, HOMO, and LUMO orbitals of all drugs; hydrophobic and aromatic surface, noncovalent interactions in the drug-receptor complexes are included in the supplementary Figures S1-S13.

## Figures and Tables

**Table 1:** The stoichiometry, electronic energy, enthalpy, and Gibbs free energy in Hartree and dipole moment (Debye) of mitoxantrone and its halogenated derivatives.

Name	Stoichiometry	Electronic energy	Enthalpy	Gibbs free energy	Dipole moment (Debye)
Mitoxantrone	C22H28N4O6	−1516.1835	−1516.1826	−1516.2838	1.5477
D1	C22H27FN4O6	−1614.8728	−1614.8719	−1614.9746	1.3831
D2	C22H27FN4O6	−1614.8711	−1614.8702	−1614.9731	2.2832
D3	C22H27ClN4O6	−1973.6278	−1973.6269	−1973.7305	2.1425
D4	C22H27ClN4O6	−1973.6169	−1973.6159	−1973.7201	5.5153
D5	C22H27BrN4O6	−4077.6078	−4077.6068	−4077.7124	1.3627
D6	C22H27BrN4O6	−4077.5965	−4077.5956	−4077.6995	4.8794
D7	C22H27IN4O6	−8405.7319	−8405.7309	−8405.8375	1.4454
D8	C22H27IN4O6	−8405.7210	−8405.7201	−8405.8257	5.3011
D9	C23H27F3N4O6	−1851.3391	−1851.3382	−1851.4476	1.6814
D10	C23H27F3N4O6	−1851.3296	−1851.3287	−1851.4370	4.5242

**Table 2:** Energy (atomic unit) of HOMO, LUMO, Gap, hardness, and softness of all drugs.

Molecules	*ε*_HOMO−1_	*ε*_HOMO_	*ε*_LUMO_	*ε*_LUMO+1_	Gap	Η (hardness)	S (softness)
Mitoxantrone	−0.2027	−0.1576	−0.0753	−0.0092	0.0822	0.0411	24.3161
D1	−0.2066	−0.1600	−0.0774	−0.0143	0.0825	0.0412	24.2248
D2	−0.2047	−0.1608	−0.0769	−0.0124	0.0838	0.0419	23.8578
D3	−0.2117	−0.1635	−0.0828	−0.0179	0.0806	0.0403	24.7892
D4	−0.2123	−0.1720	−0.0872	−0.0225	0.0847	0.0423	23.5932
D5	−0.2078	−0.1612	−0.0802	−0.0149	0.0810	0.0405	24.6883
D6	−0.2138	−0.1767	−0.0887	−0.0259	0.0879	0.0439	22.7298
D7	−0.2080	−0.1614	−0.0806	−0.0455	0.0808	0.0404	24.7494
D8	−0.2149	−0.1791	−0.0903	−0.0308	0.0888	0.0444	22.5098
D9	−0.2109	−0.1630	−0.0830	−0.0217	0.0799	0.0399	25.0250
D10	−0.2132	−0.1734	−0.0887	−0.0266	0.0846	0.0423	23.6211

**Table 3:** Binding energy and nonbonding interaction of mitoxantrone and its derivatives.

Compound	Docking against human topoisomerase II alpha (TOP2A)								
	Binding energy (kcal mol^−1^)	H-bond		Halogen bond		Hydrophobic interaction		Electrostatic interaction	
		Amino acid-ligand atom	Distance (Å)	Amino acid-ligand atom	Distance (Å)	Amino acid-ligand atom	Distance (Å)	Amino acid-ligand atom	Distance (Å)
Mitoxantrone (D)	−9.2	Lys723 [N-H⋯O] Ile856 [C-O⋯H]^*∗*^ Asp710 [C-O⋯H]^*∗*^ Ile856 [C-O⋯H]^*∗*^ Asp710 [C-O⋯H]^*∗*^	2.2693 3.0872 2.9119 3.0135 2.2831			Lys723 [alkyl-pi] Lys723 [alkyl-pi]	4.8912 4.8521		

D1	−9.5	Glu712 [C-O⋯H] Glu839 [C-O⋯H]^*∗*^ His1005 [C-O⋯H]^*∗*^ Val1006 [C-O⋯H]^*∗*^ Glu839 [C-O⋯H]^*∗*^ His1005 [C-O⋯H]^*∗*^ Glu712 [C-O⋯H ]^*∗*^	2.8309 2.4579 2.8559 2.1817 2.7366 2.7720 2.6202	Ile715 [C-O⋯F]	3.6260	Phe1003 [pi-pi staked]	4.7087	Glu839 [anion-pi]	3.9962

D2	−9.4	Lys723 [N-H⋯O] Asp710 [C-H⋯O]^*∗*^ Asp710 [C-H⋯O]^*∗*^	2.2143 2.9100 2.2783			Lys723 [alkyl-pi] Lys723 [alkyl-pi]	4.8686 4.8569		

D3	−9.8	Ser709 [C-H⋯N]^*∗*^ Ser756 [C-H⋯Cl]^*∗*^ Asp545 [C-O⋯H]^*∗*^ His759 [N⋯H]^*∗*^ Glu839 [C-O⋯H]^*∗*^ Gln544 [C-O⋯H]^*∗*^	2.3741 2.8450 2.9559 2.6541 2.1800 2.2268			Val836 [alkyl-Cl] Pro724 [alkyl-pi] Val836 [alkyl-pi] His758 [pi-Cl]	4.5719 4.5000 5.2539 5.3149	Lys728 [cation-pi] Asp831 [anion-pi]	2.2629 3.5500

D4	−10.3	Asn770 [N-H⋯O] Asn779 [C-O⋯H-O] Ile856 [C-O⋯H]^*∗*^ Ile856 [C-O⋯H]^*∗*^ Asp710 [C-O⋯H]^*∗*^ Ile856 [C-O⋯H]^*∗*^	2.3508 2.4357 2.9606 3.0344 2.5335 2.4966			Lys723 [sigma-pi] Lys723 [alkyl-pi]	2.7069 4.9847	Lys723 [cation-pi]	2.7975

D5	−9.3	Lys723 [N-H⋯O] Ile856 [C-O⋯H]^*∗*^ Asp710 [C-O⋯H]^*∗*^ Ile856 [C-O⋯H]^*∗*^ Asp710 [C-O⋯H]^*∗*^	2.0085 2.9432 2.8721 2.9262 2.3241			Lys723 [alkyl-pi] Lys723 [alkyl-pi] Tyr757 [pi-Br]	4.6518 4.9056 4.8900		

D6	−9.9	Asn770 [C-O⋯H-N] Asn770 [O⋯H-N] Gly852 [C-O⋯H-O] Ile856 [C-O⋯H]^*∗*^ Asn710 [C-O⋯H]^*∗*^ Ile856 [C-O⋯H]^*∗*^	2.3828 2.6450 2.7306 2.8943 2.4152 2.6609			Lys723 [sigma-pi] Lys723 [alkyl-pi]	2.6360 5.1763	Lys723 [cation-pi] Lys723 [cation-pi]	3.5014 2.9085

D7	−9.5	Glu839 [C-O⋯H-N] Val1006 [C-O⋯H-N] Ser717 [O⋯H] Glu839 [C-O⋯H]^*∗*^ Val1006 [C-O⋯H]^*∗*^ His1005 [C-O⋯H]^*∗*^	3.0102 2.9825 2.6782 2.7621 2.2233 3.0479			Phe1003 [pi-pi stacked] Pro724 [alkyl-I] Phe1003 [pi-I]	5.0840 4.6167 4.7419	Glu839 [anion-pi]	3.7952

D8	−9.7	Asn770 [C-O⋯H-N] Asn770 [O⋯H-N] Gly852 [C-O⋯H] Ile856 [C-O⋯H]^*∗*^ Asn710 [C-O⋯H]^*∗*^ Ile856 [C-O⋯H]^*∗*^	2.3318 2.3759 2.8141 2.8758 2.6189 2.4101			Lys723 [alkyl-pi] Lys723 [alkyl-pi]	4.2645 5.3858	Lys723 [cation-pi] Lys723 [cation-pi]	3.5624 2.7612

D9	−10.3	Lys723 [N-H⋯O-C] Ile856 [C-O⋯H]^*∗*^ Asp710 [C-O⋯H]^*∗*^ Ile856 [C-O⋯H]^*∗*^ Asp710 [C-O⋯H]^*∗*^	2.0587 2.9275 2.9210 2.8580 2.3996	Gly725 [N-H⋯F] Gly725 [N-H⋯F] Gly725 [C-H⋯F]	2.9348 2.6339 2.2421	Tyr757 [pi-alkyl] Lys723 [alkyl-pi] Lys723 [alkyl-pi]	5.1734 4.7419 4.9257		

D10	−10.0	Arg713 [N-H⋯O-C] Arg713 [N-H⋯O] Lys723 [N-H⋯F] Ser763 [C-O⋯N] Arg929 [N-H⋯O] Arg929 [N-H⋯O] His759 [C-O⋯H]^*∗*^	2.4985 2.3481 2.2497 2.4413 2.9278 2.8935 2.8837	Arg713 [C-O⋯F]	3.3749	Lys723 [alkyl-alkyl] Lys723 [alky-pi] Ille856 [alkyl-pi]	4.2984 5.4568 5.0991		

^*∗*^Nonconventional hydrogen bond.
