# Supplementary material for: Inhibition of DNA Topoisomerase Type IIα (TOP2A) by Mitoxantrone and Its Halogenated Derivatives: A Combined Density Functional and Molecular Docking Study
Source: Biomed Res Int. 2016 Feb 15;2016:6817502. doi: 10.1155/2016/6817502 (PMC4754470; doi:10.1155/2016/6817502)
Supplement: Supplementary file 1 — The binding pocket of the protein; dipole moment, partial charge, HOMO, and LUMO orbitals of all drugs; hydrophobic and aromatic surface, noncovalent interactions in the drug-receptor complexes are included in the supplementary Figures S1-S13. [file 6817502.f1.docx]

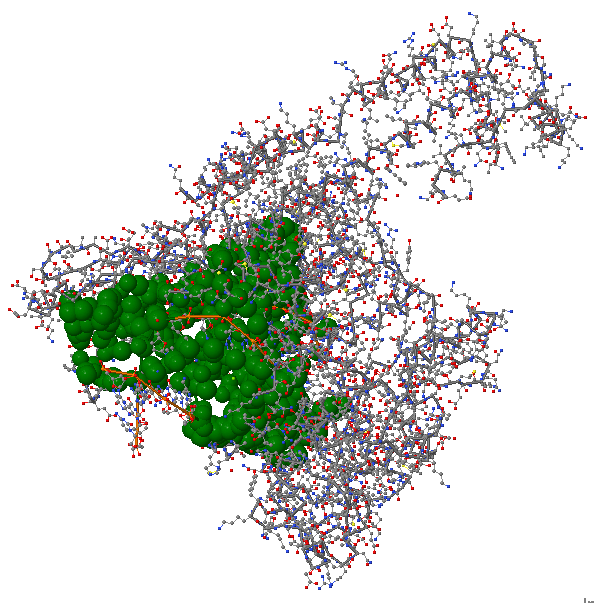

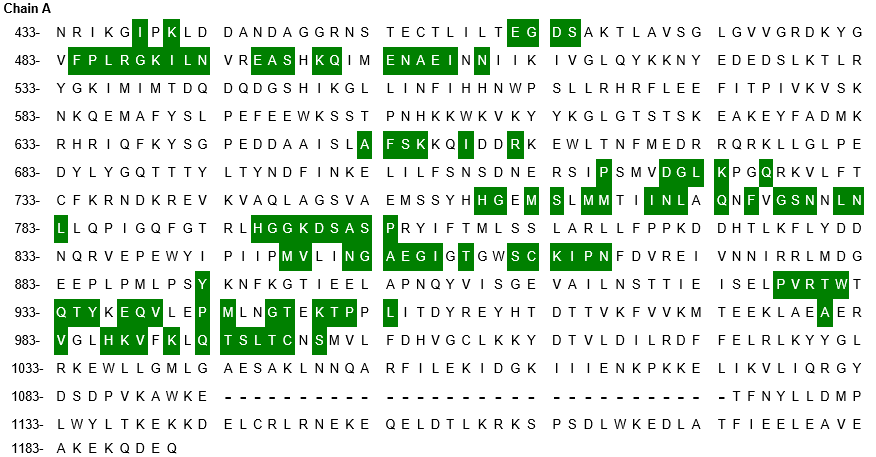


**Figure S1:** The binding pocket of TOP2A and its amino acid residues.


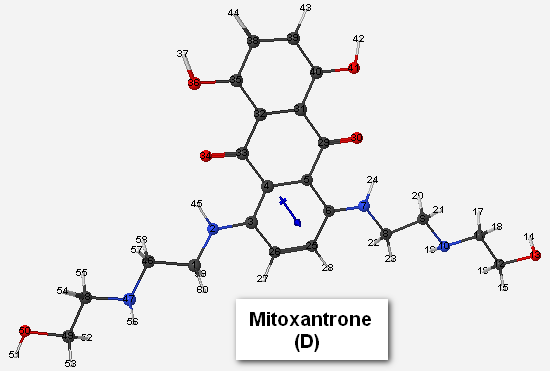

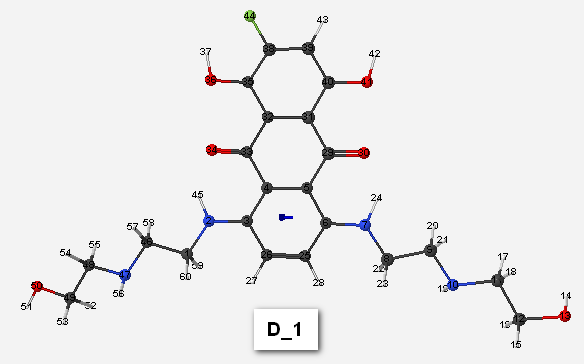

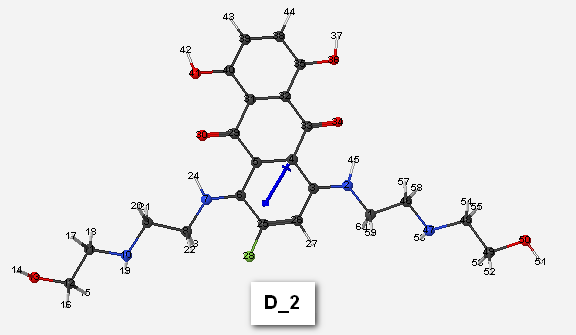

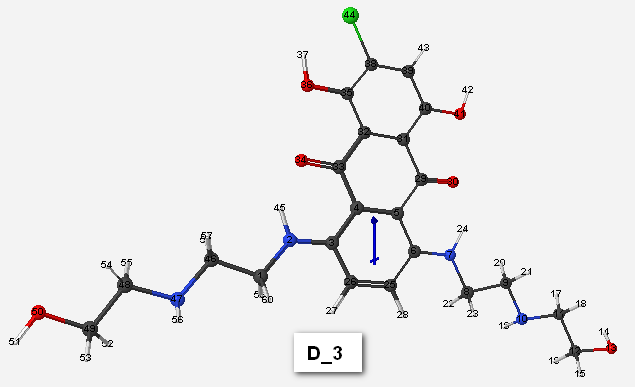

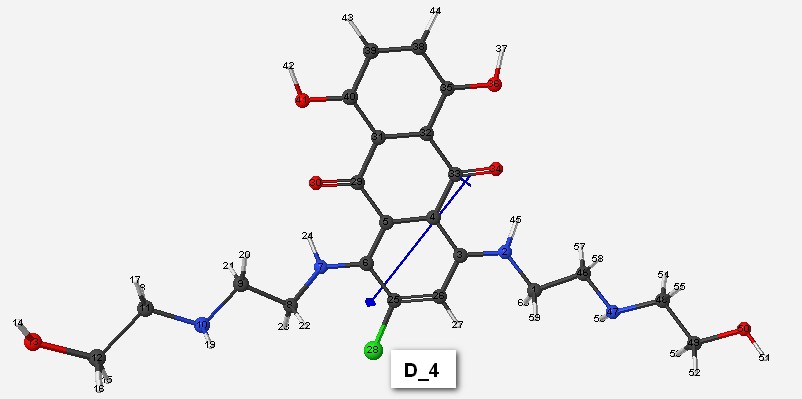

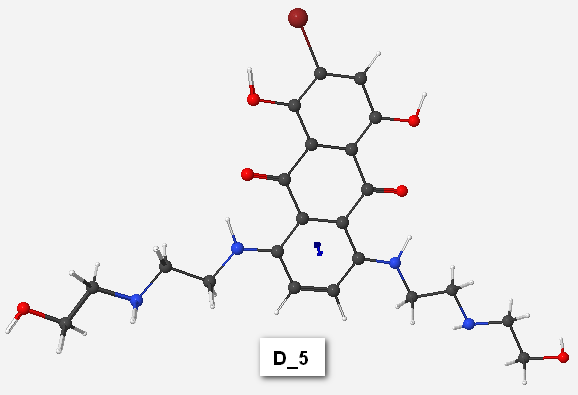

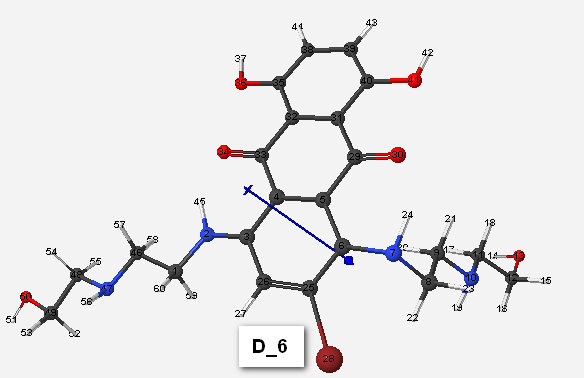

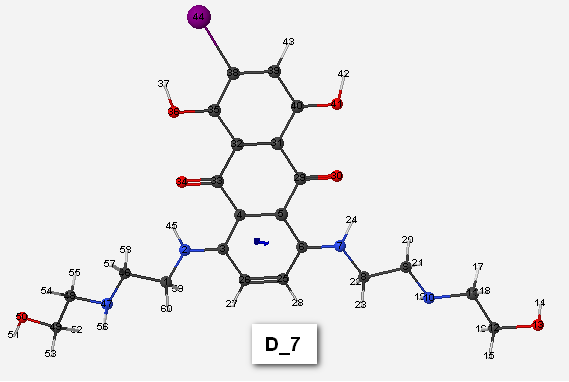

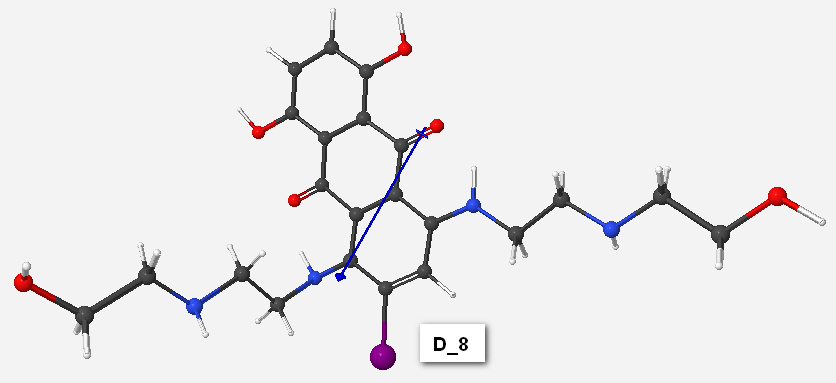

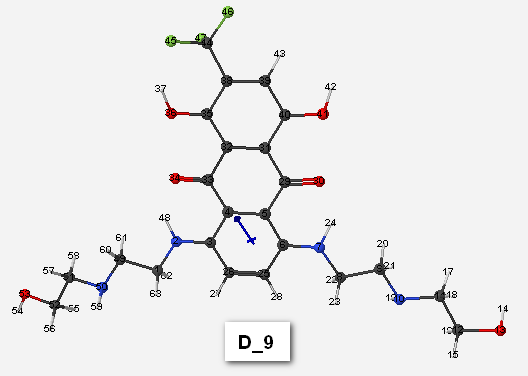

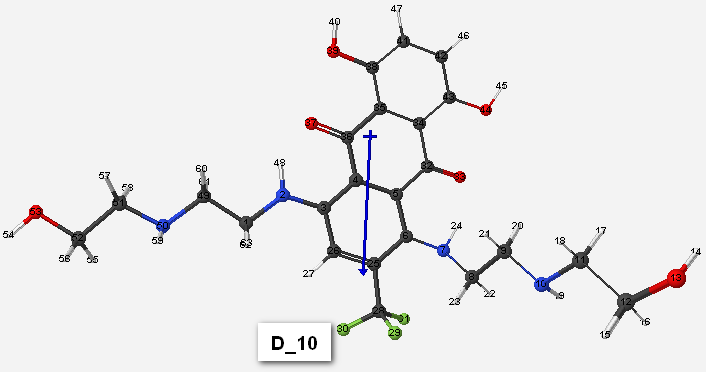


**A: Dipole moment of different drugs.**


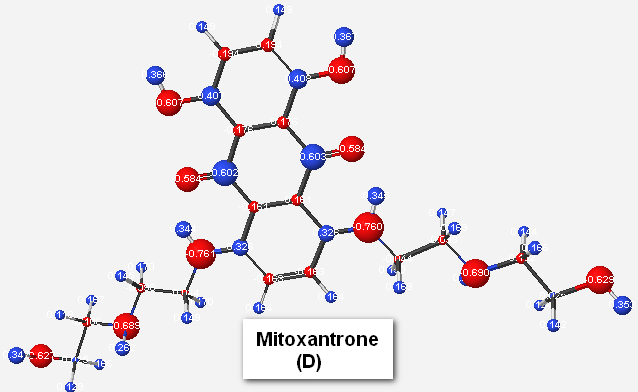

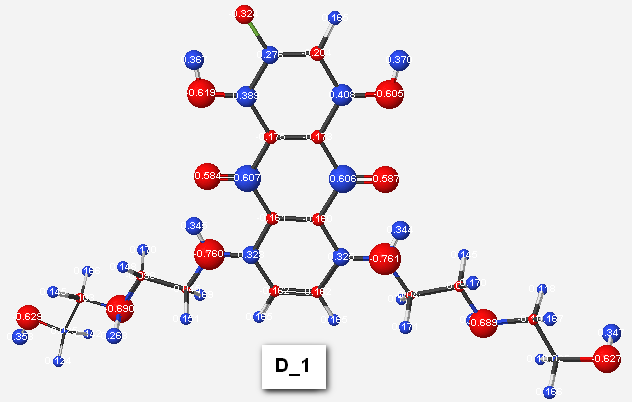

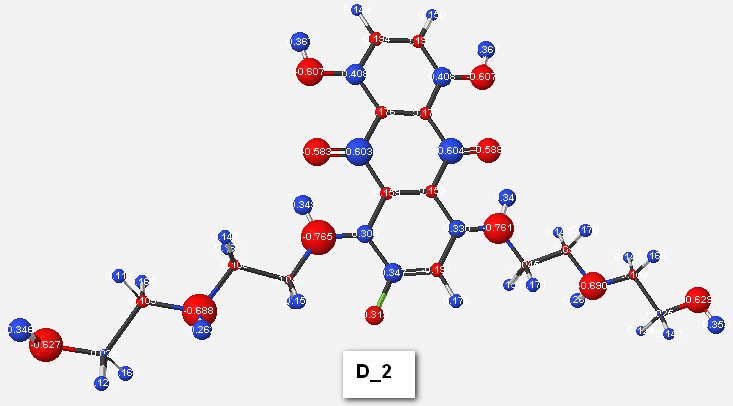

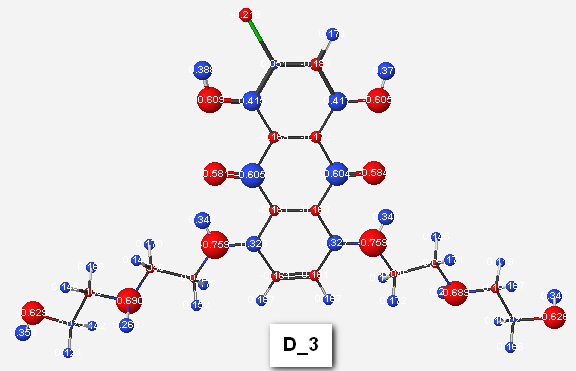

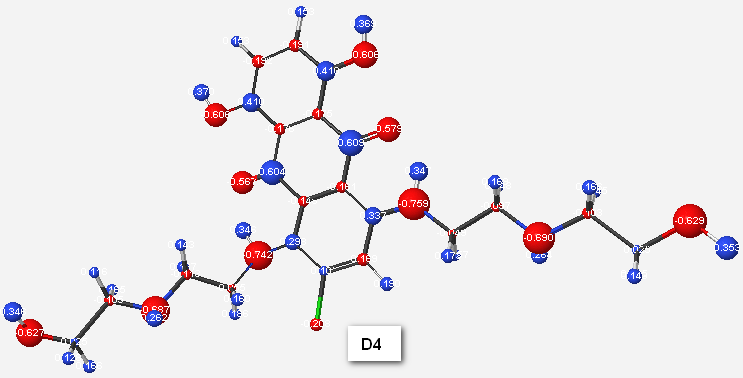

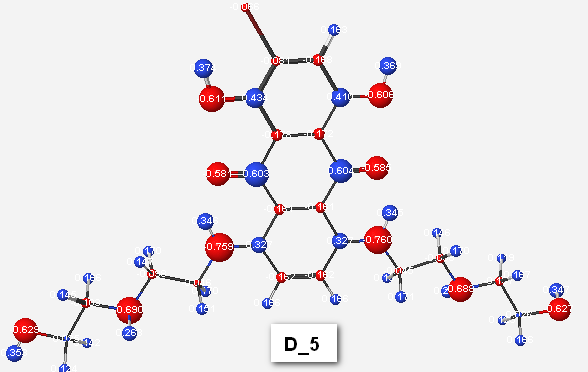

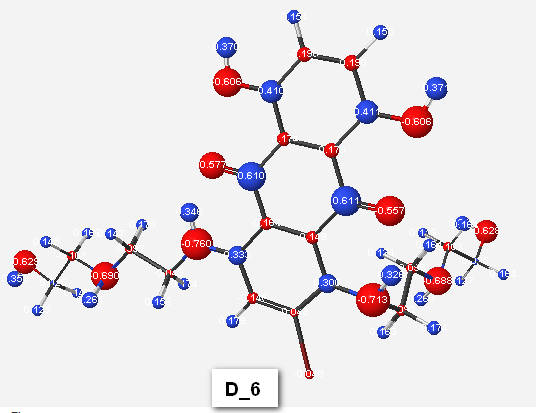

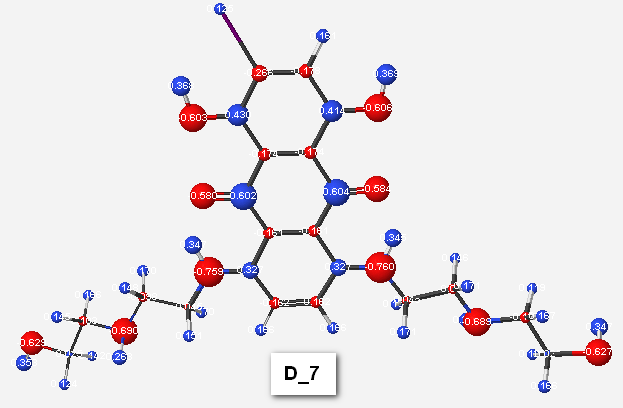

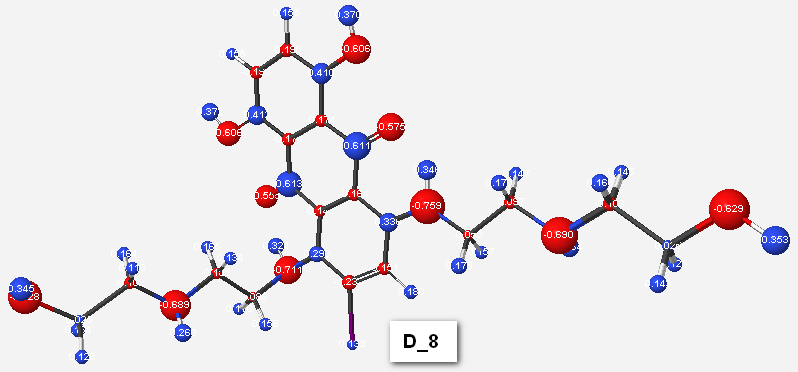

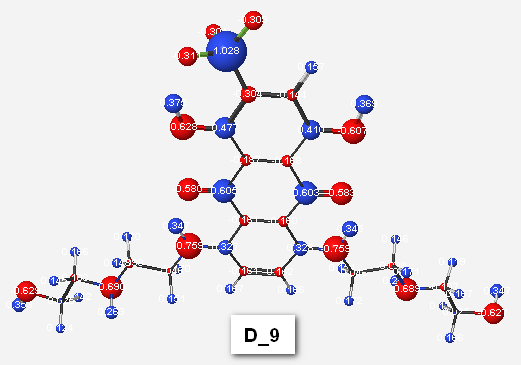

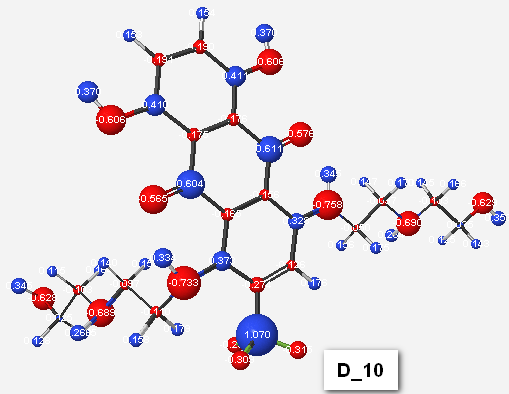


**B: Partial charges on atoms of all drug molecules.**

**Figure S2: Dipole moment and partial charge of all drugs.**


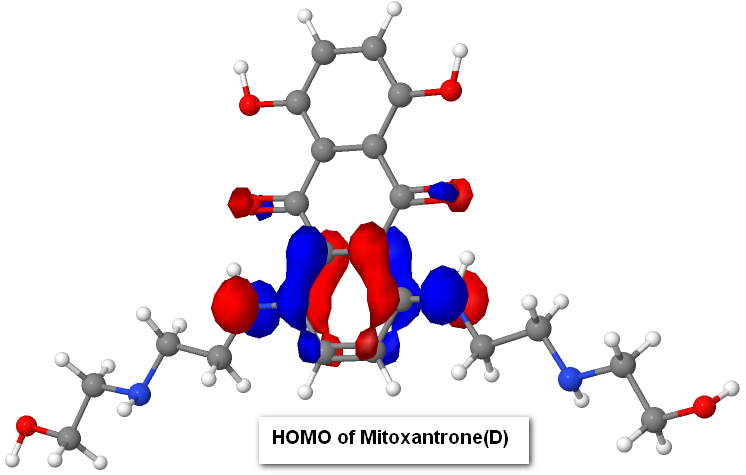

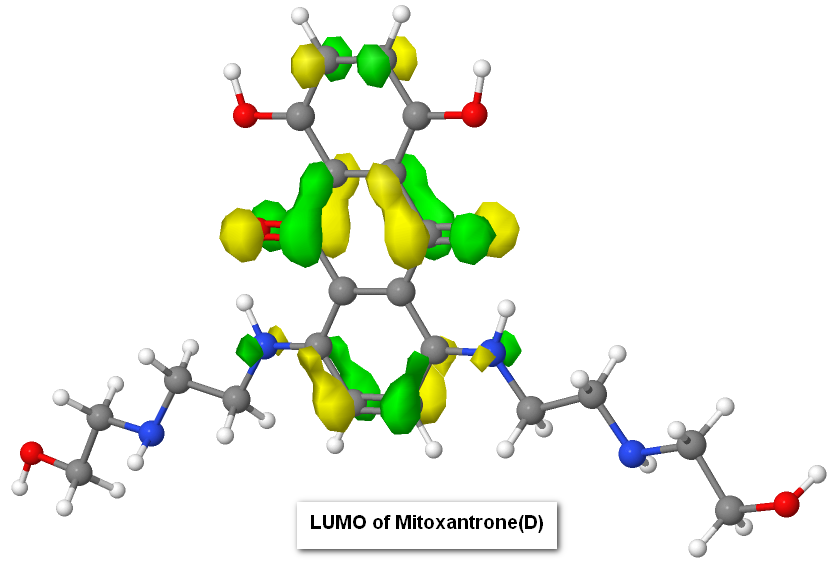


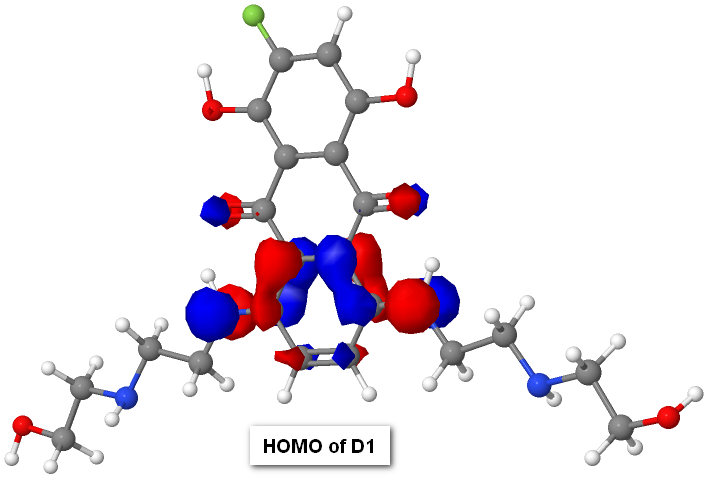

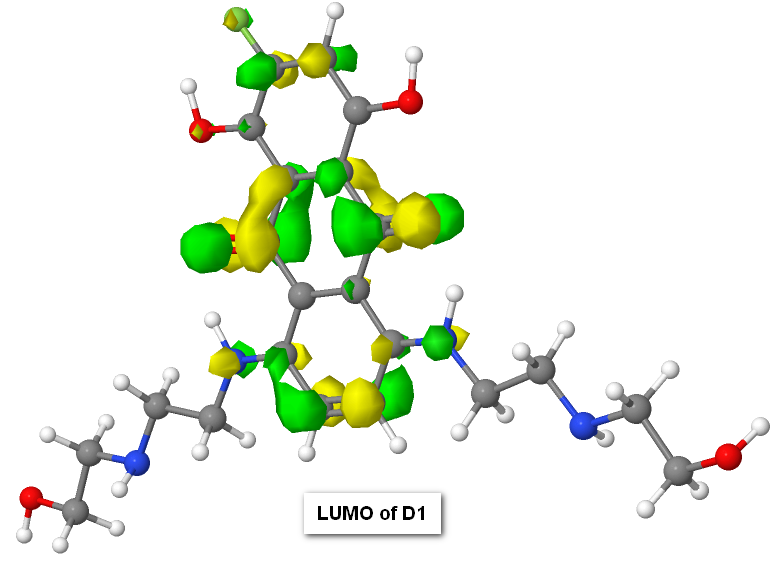


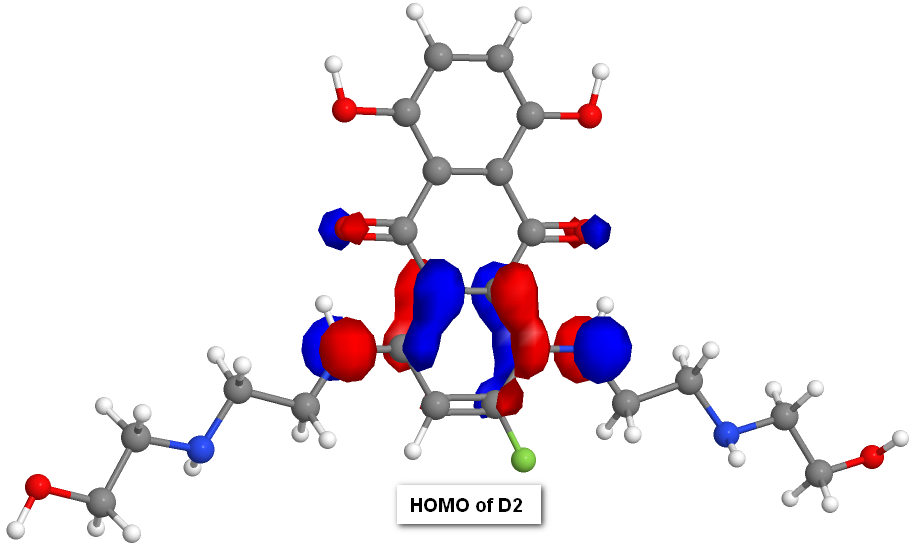

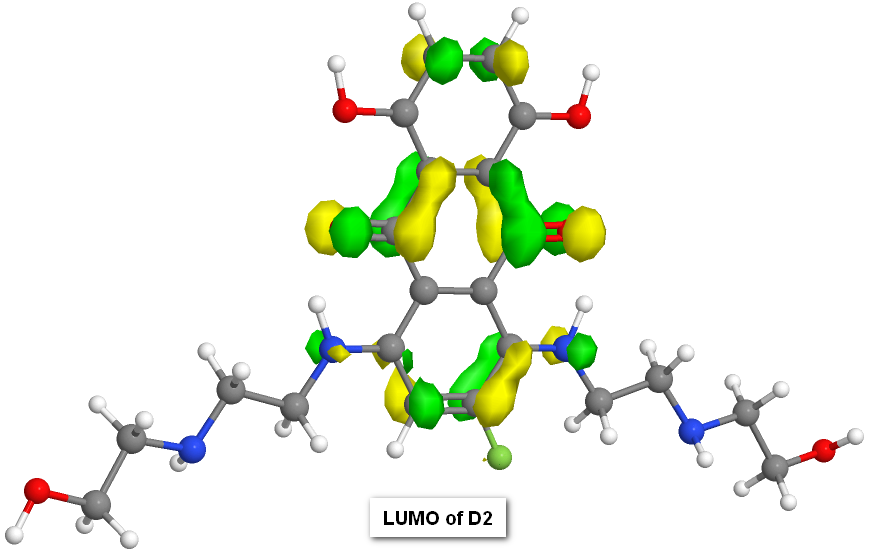


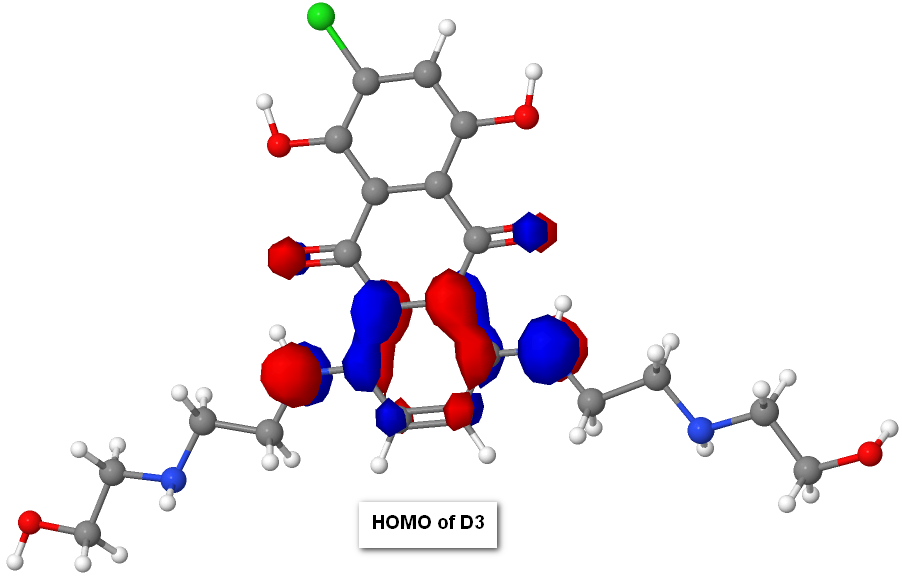

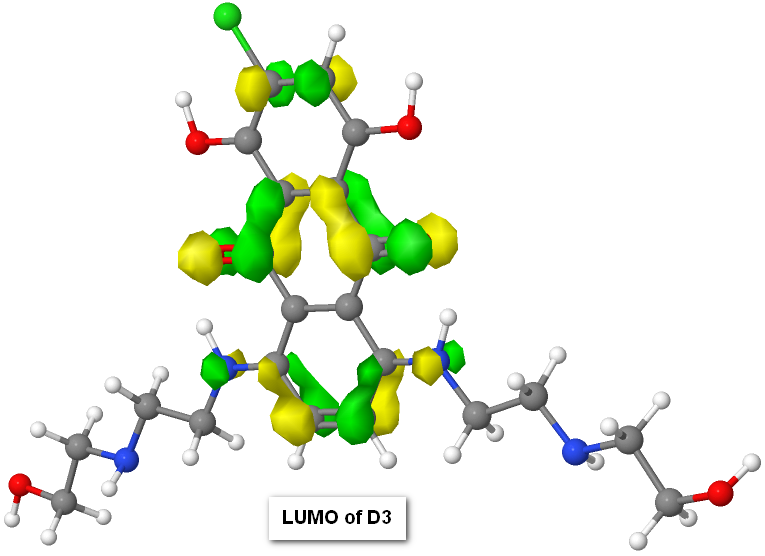


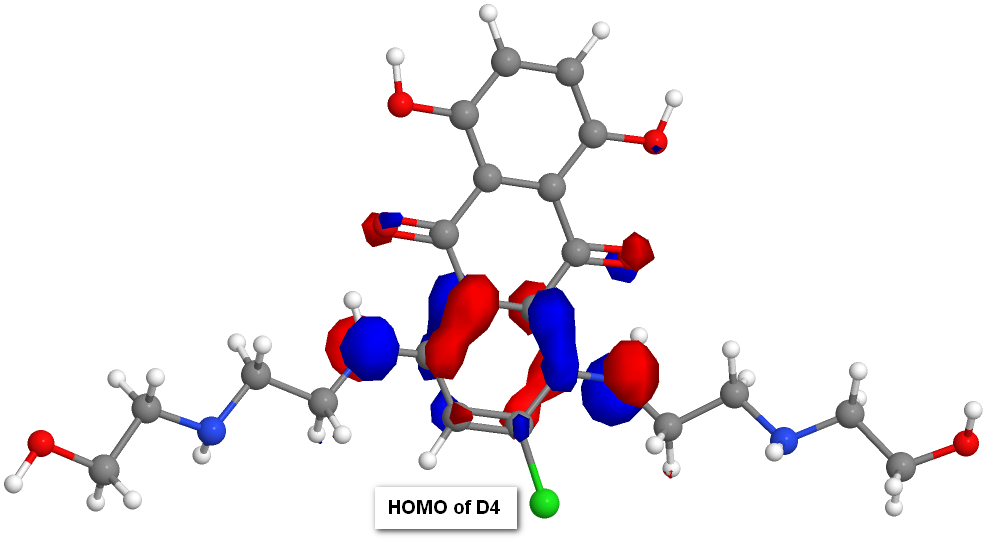

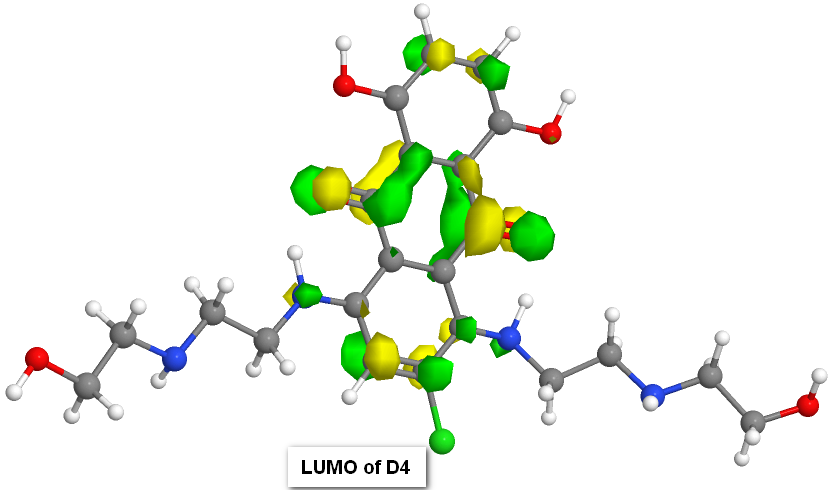


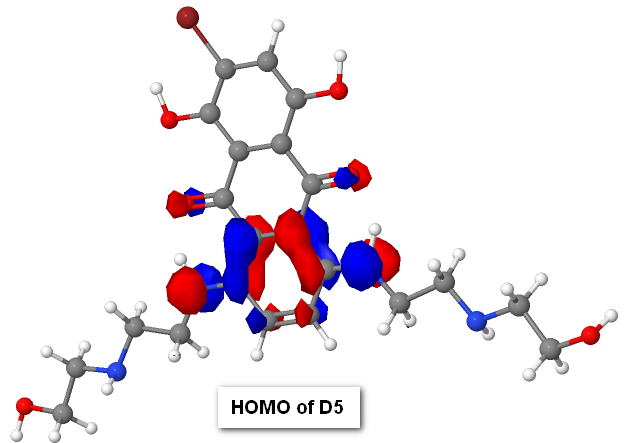


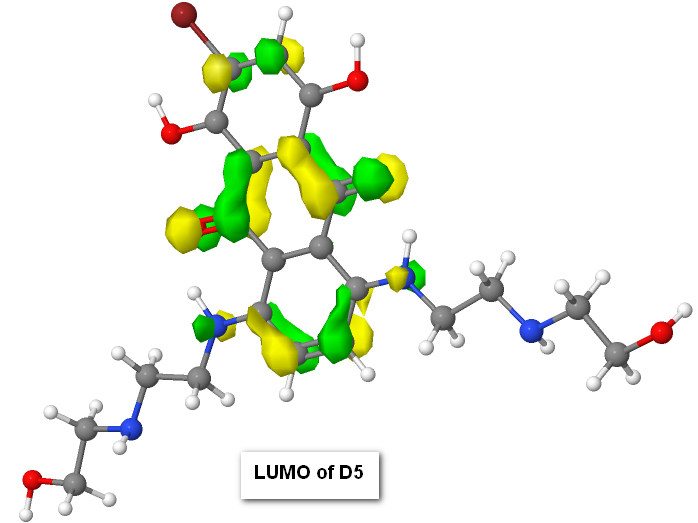


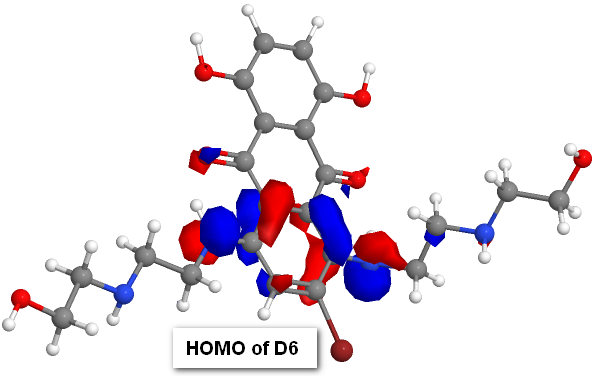

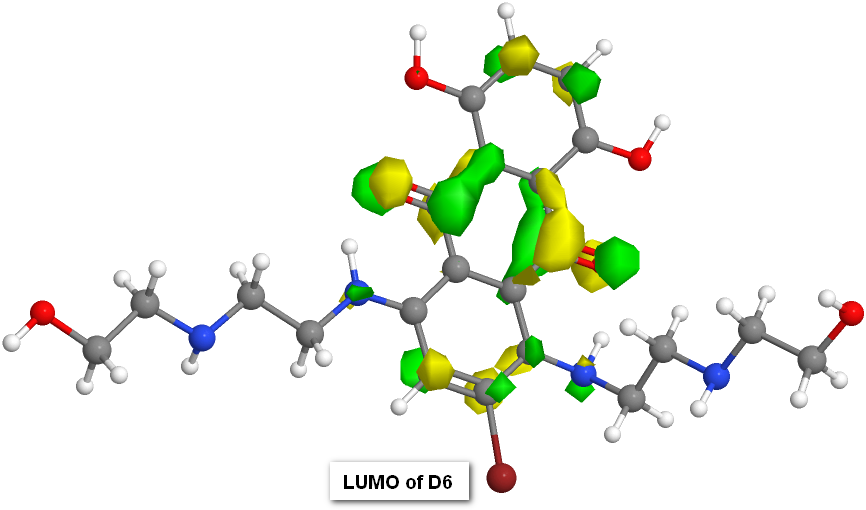


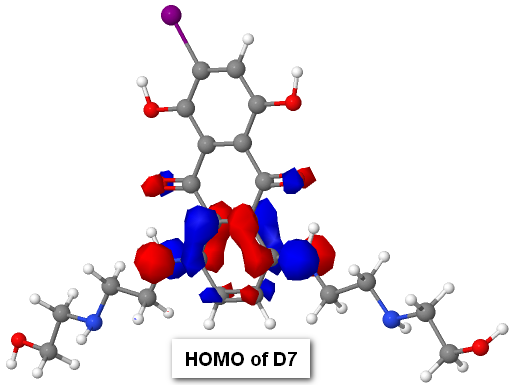

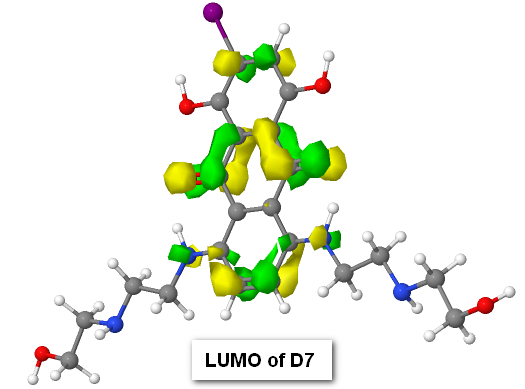


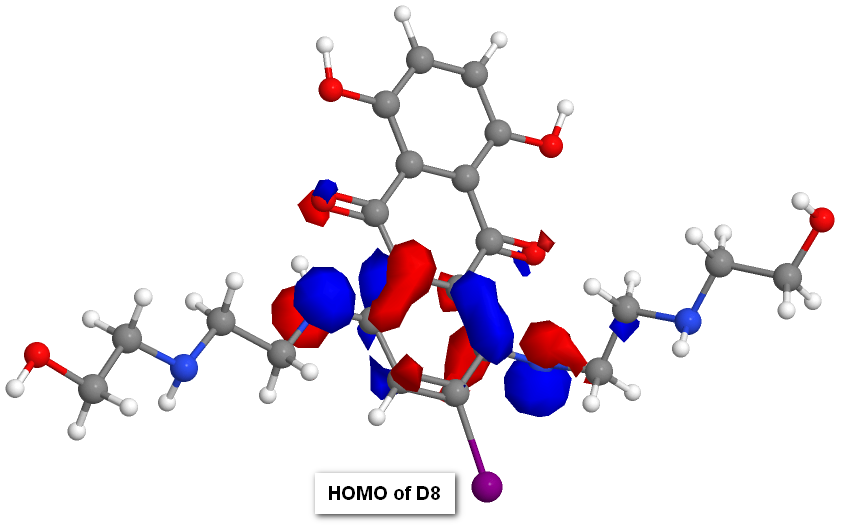

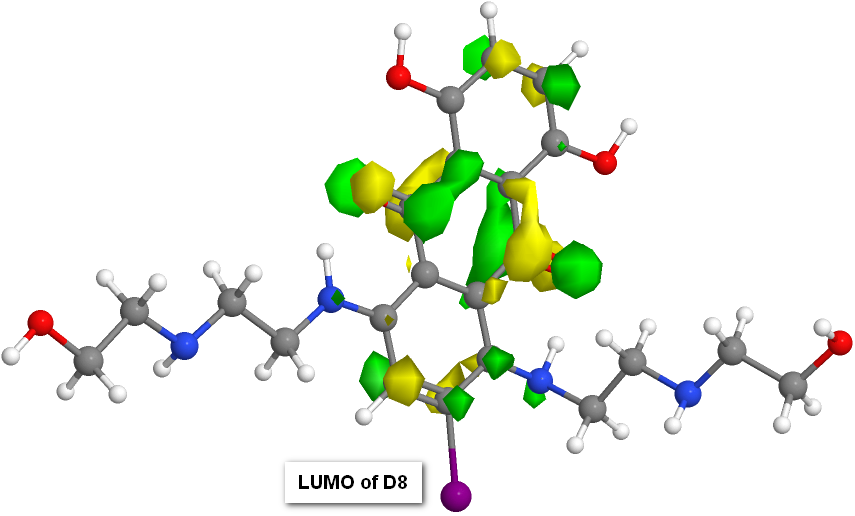


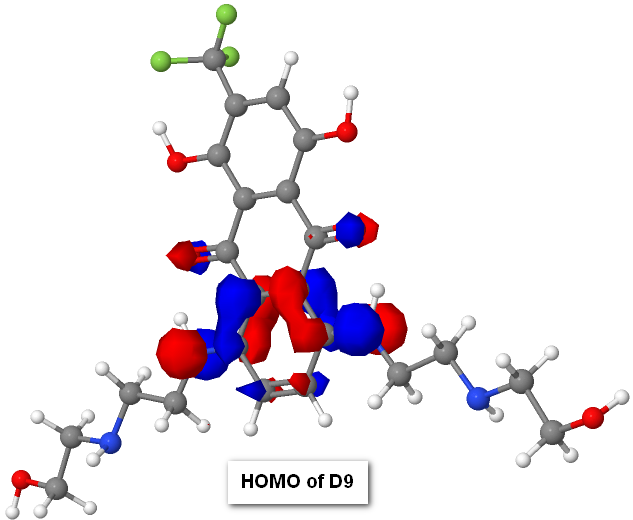


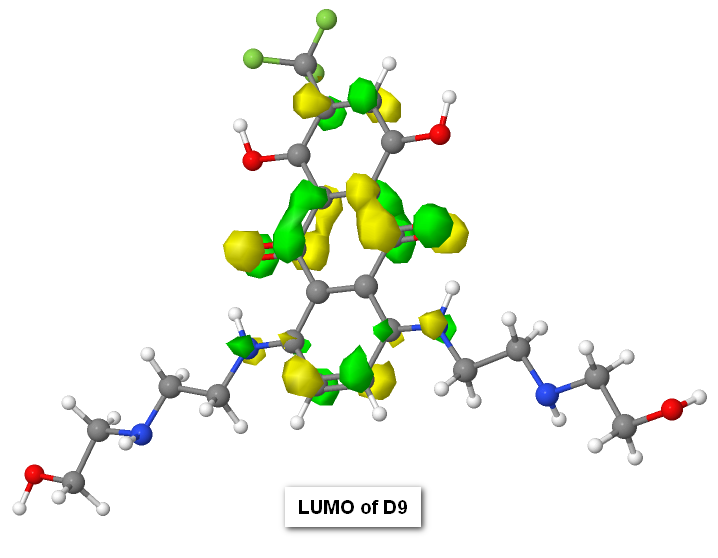


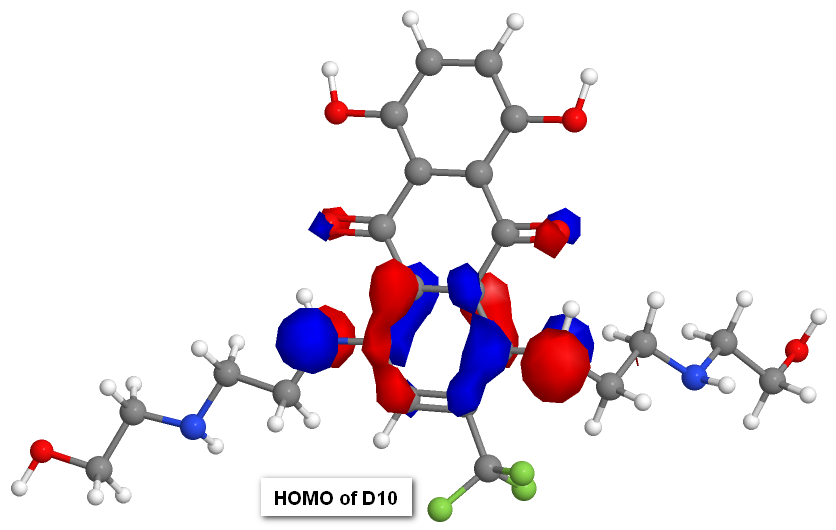


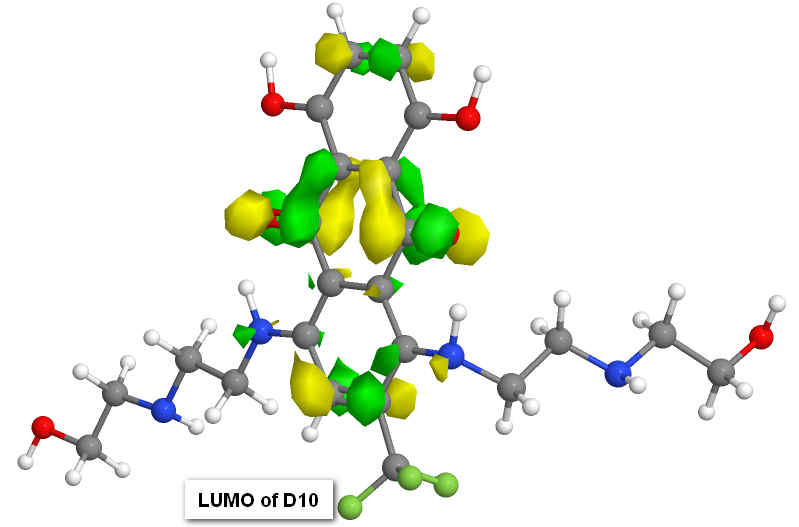


**Figure S3: HOMO and LUMO orbitals of all drugs.**

**
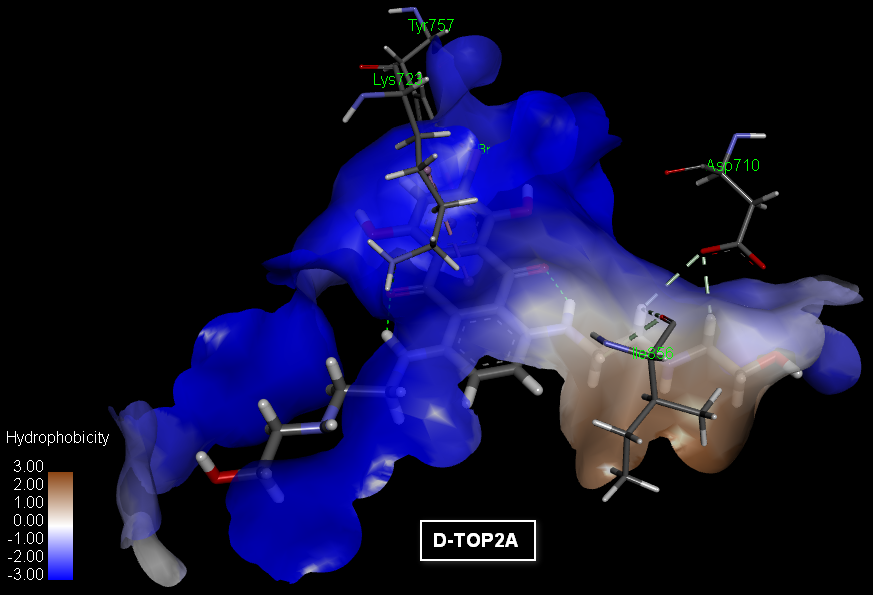

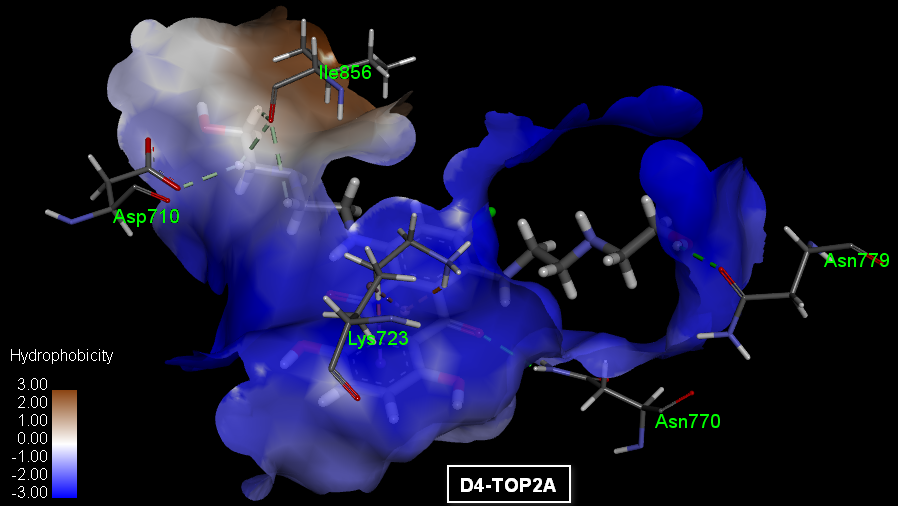

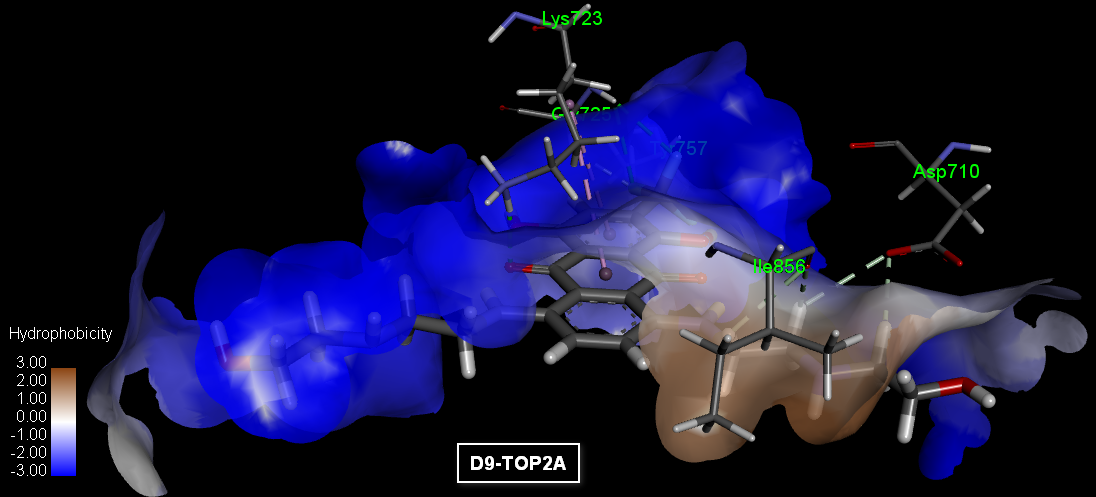

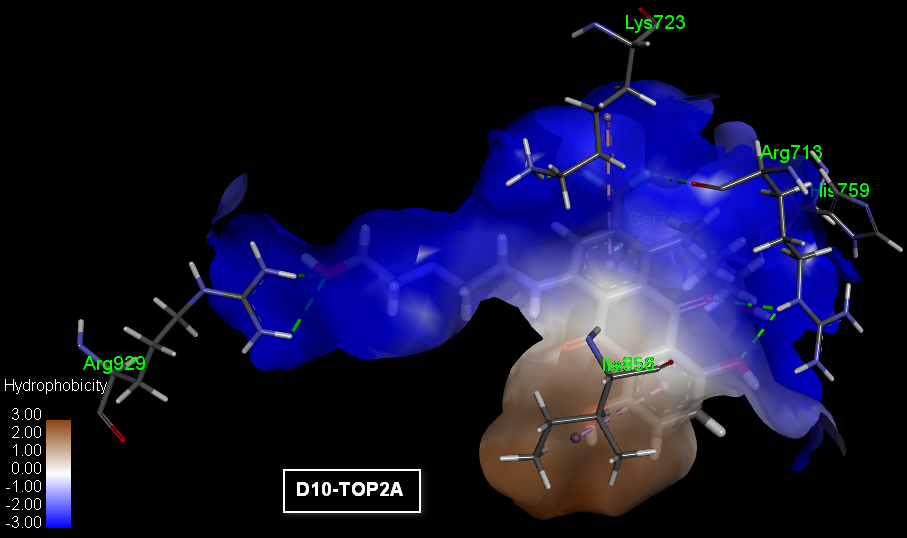
**

**Figure S4:** Hydrophobic surface of TOP2A with D, D4, D9, and D10.


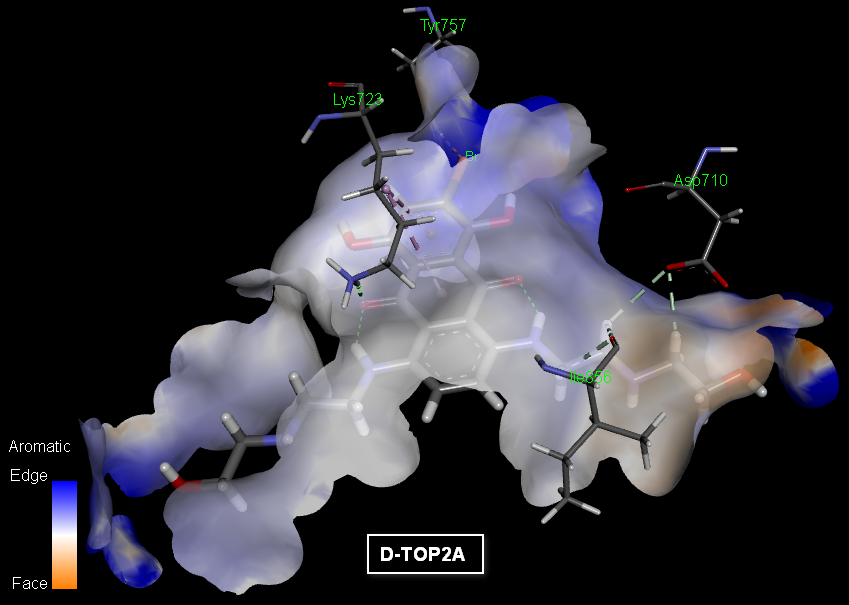

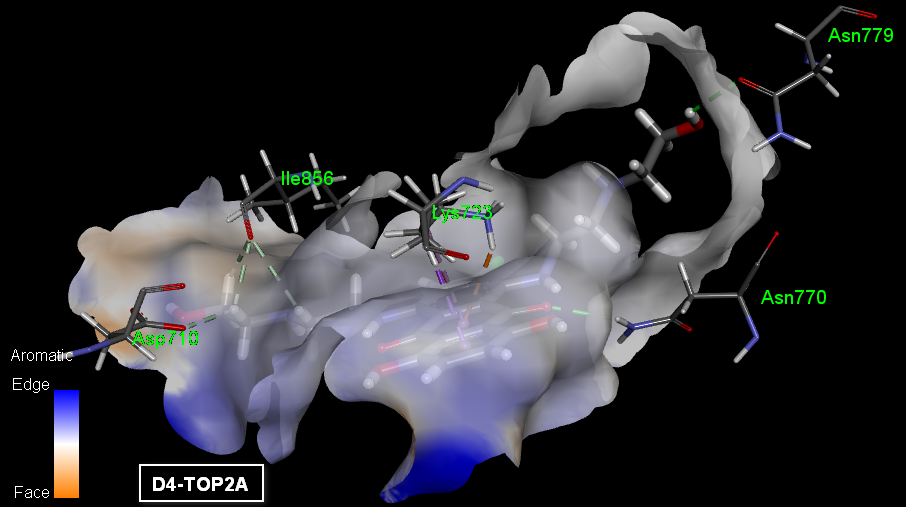


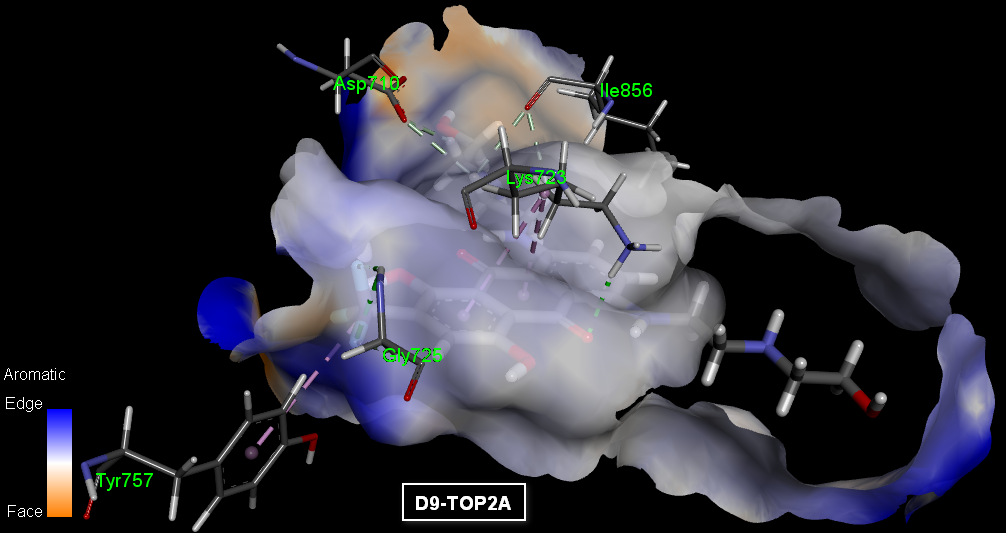

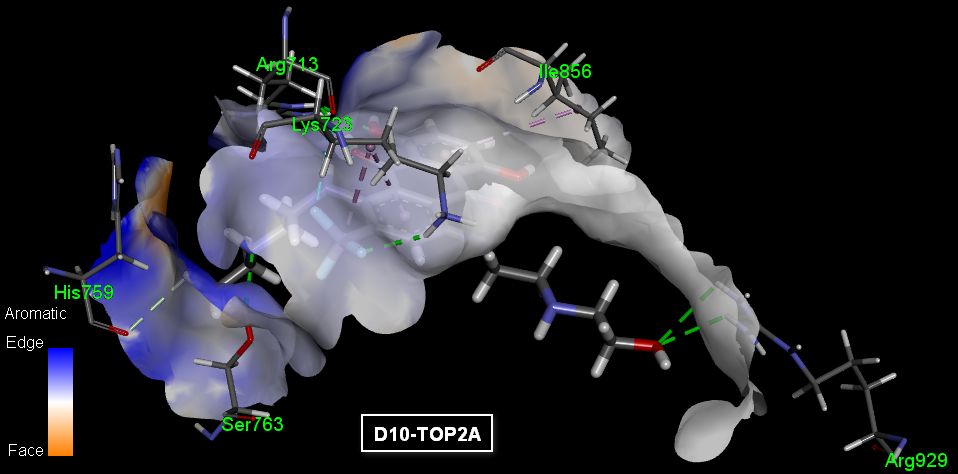


Figure S5: Aromatic surface of TOP2A with D, D4, D9, and D10.

**
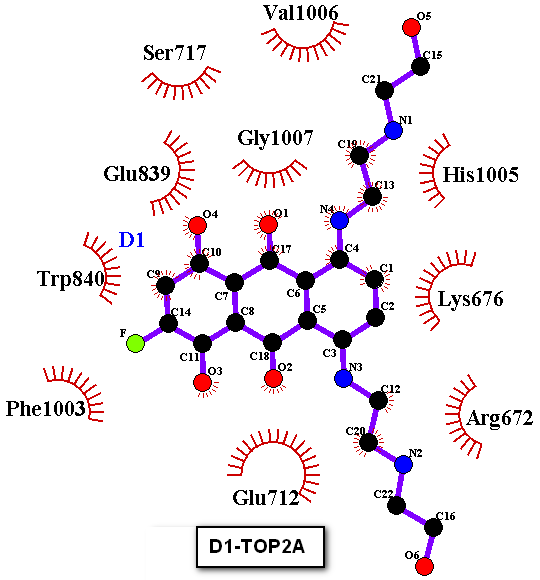

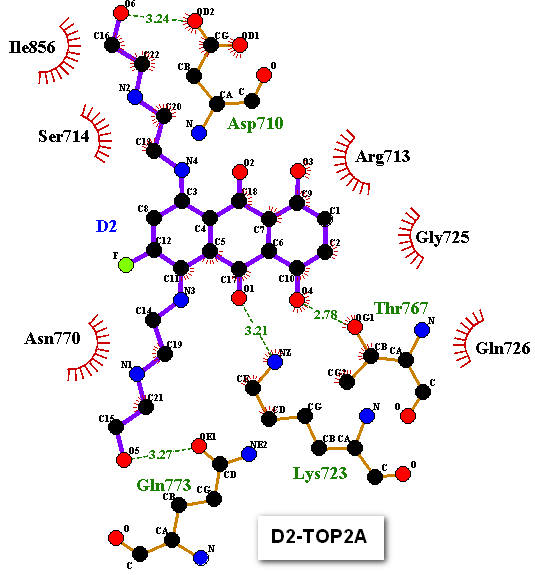

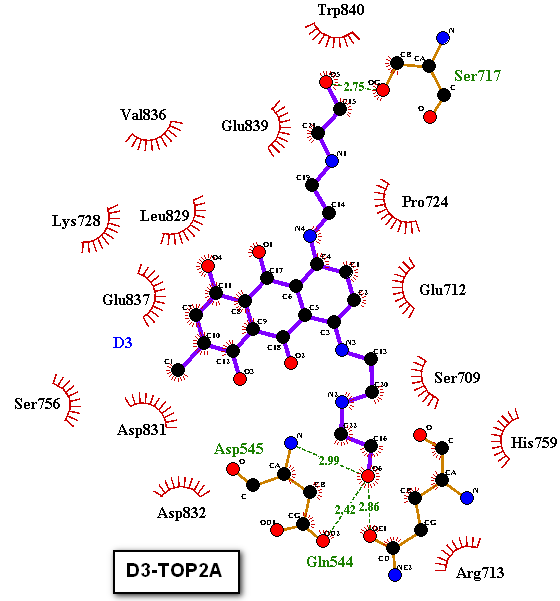

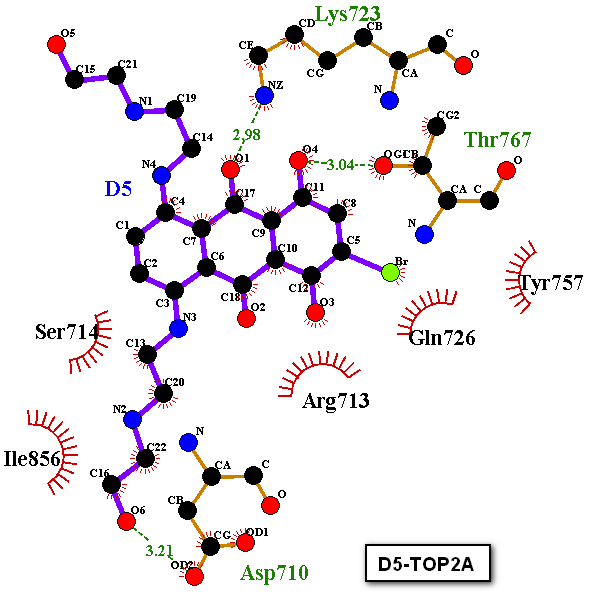

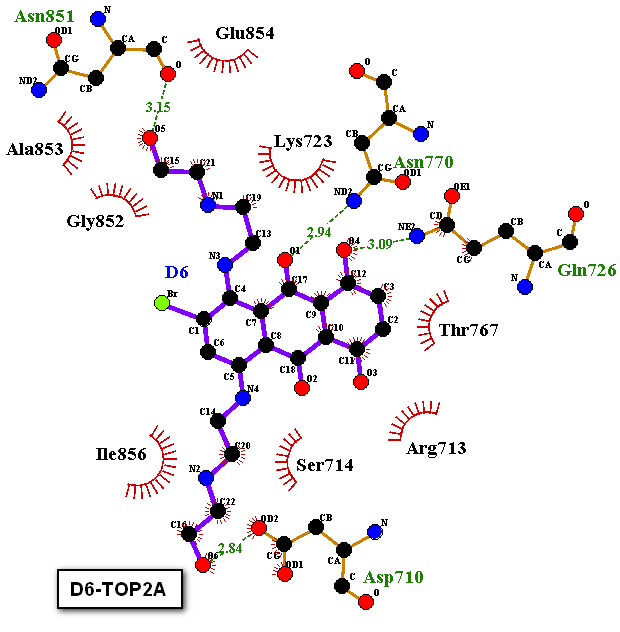

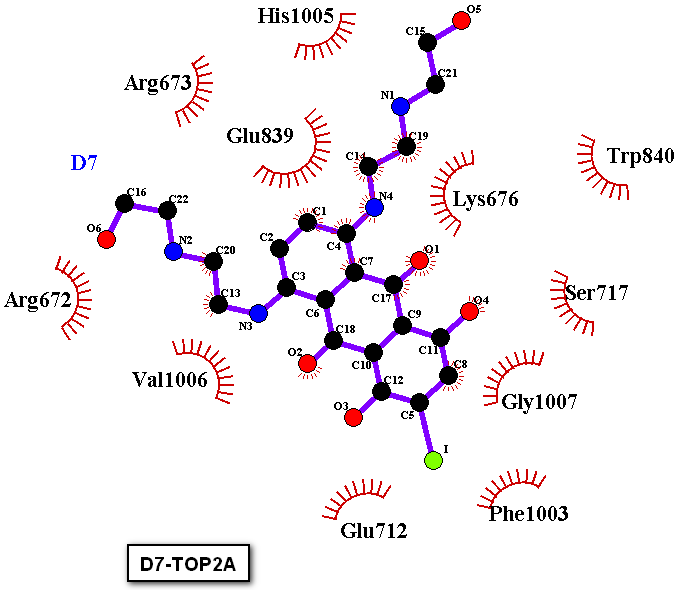

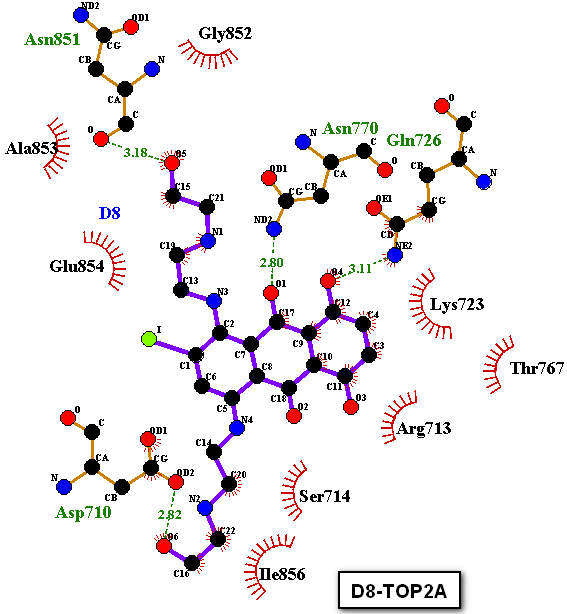
**

**Figure S6:** D1, D2, D3, D5, D6, D7, and D8 interactions with surrounding residues of TOP2A generated by LigPlus.


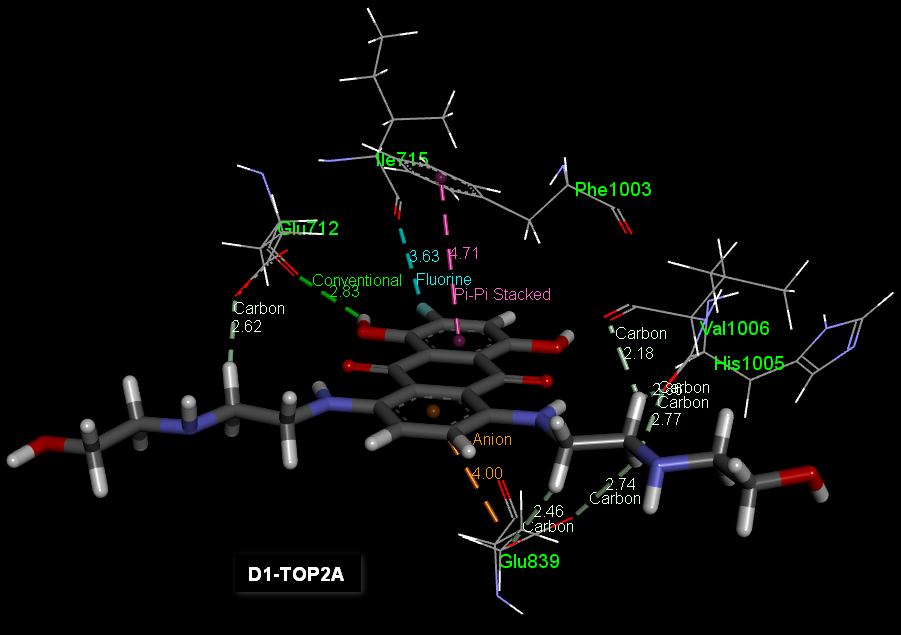


**Figure S7**: Non-bonded interactions in D1-TOP2A complex.


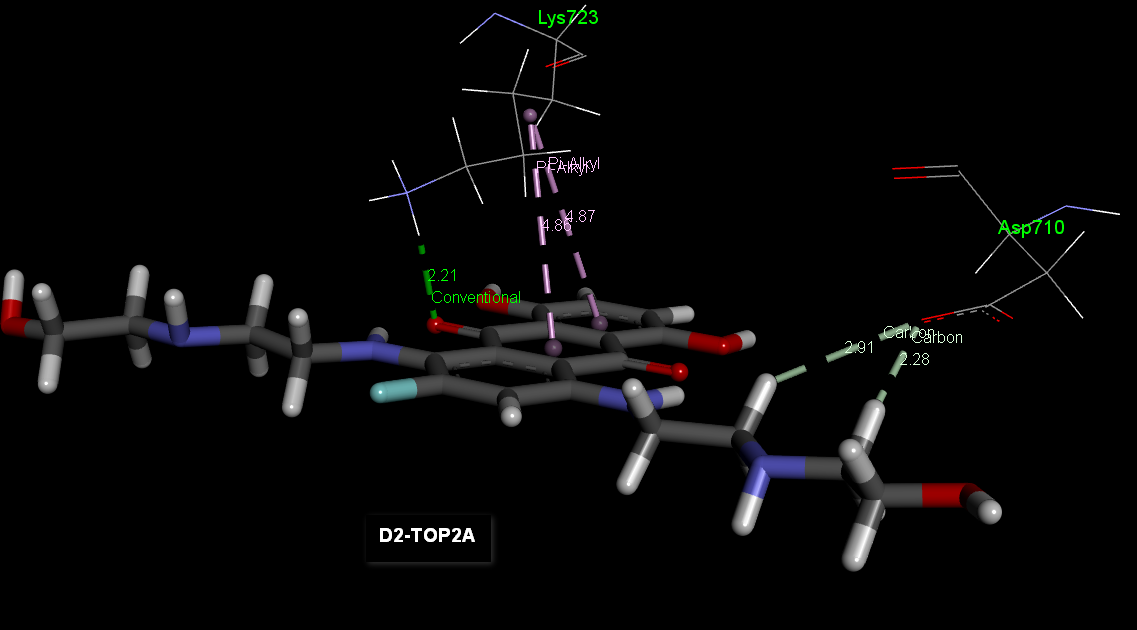


**Figure S8:** Non-bonded interactions in D2-TOP2A complex.


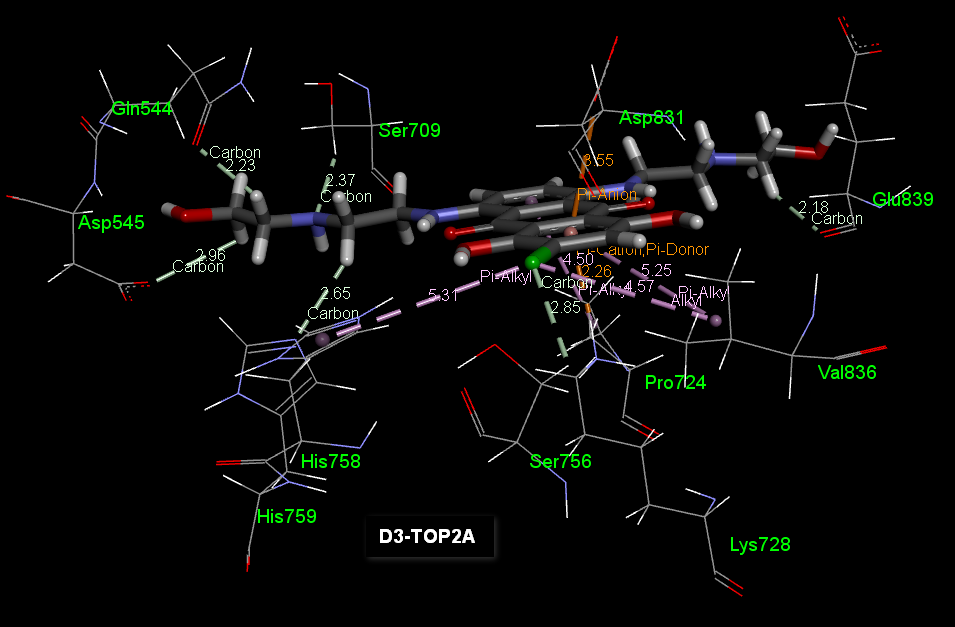


**Figure S9:** Non-bonded interactions in D3-TOP2A complex.


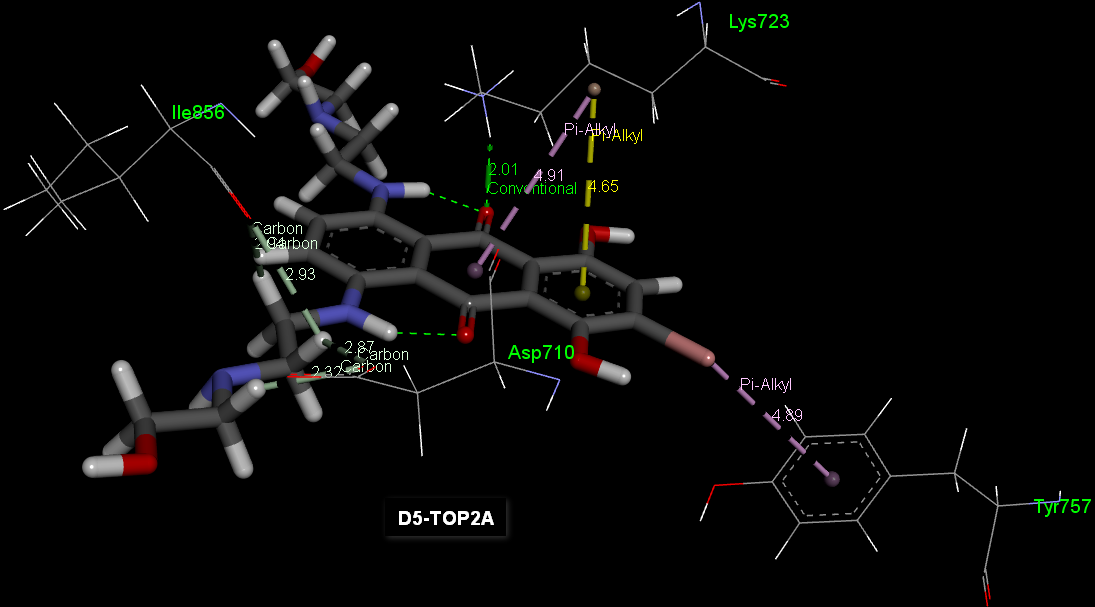


**Figure S10:** Non-bonded interactions in D5-TOP2A complex.


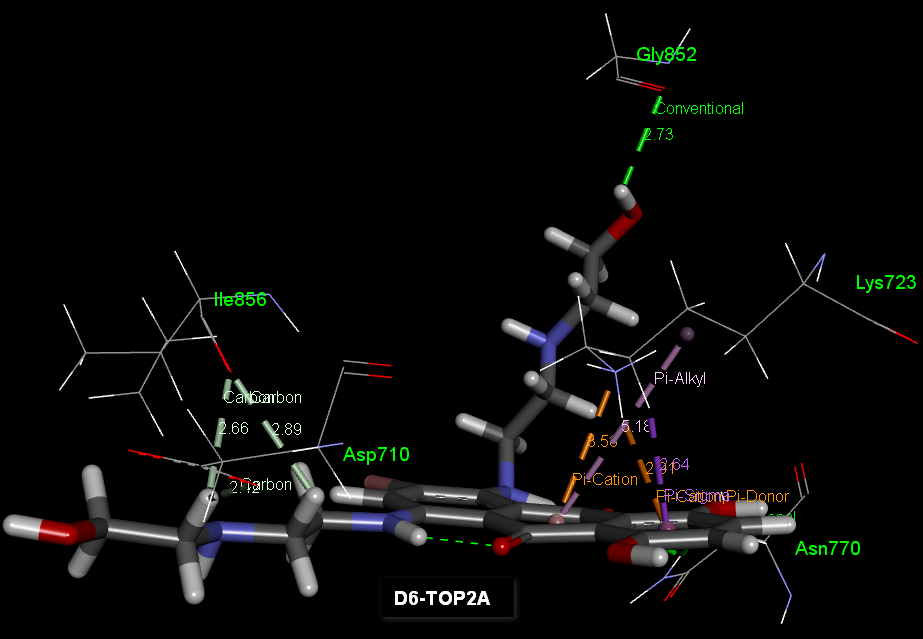


**Figure S11:** Non-bonded interactions in D6-TOP2A complex.


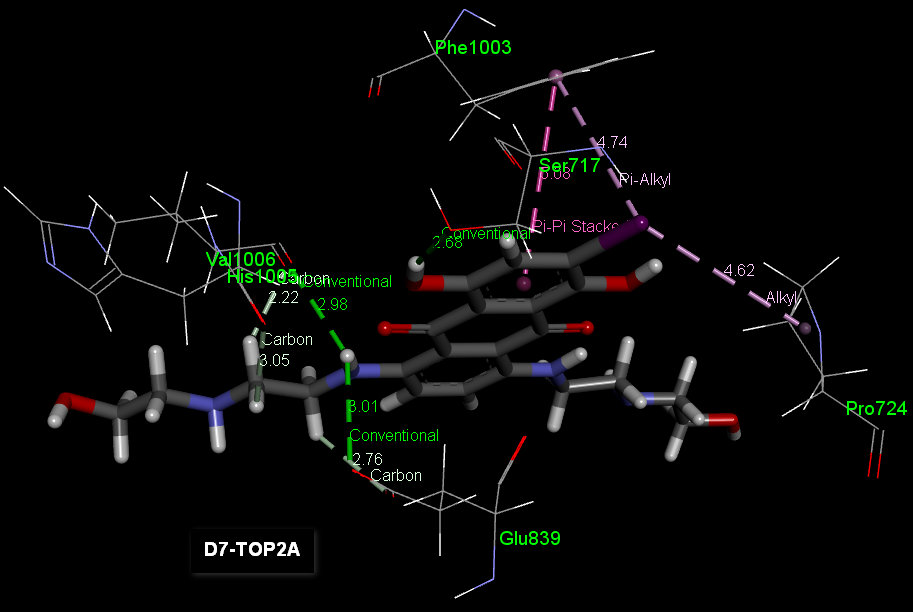


**Figure S12:** Non-bonded interactions in D7-TOP2A complex.


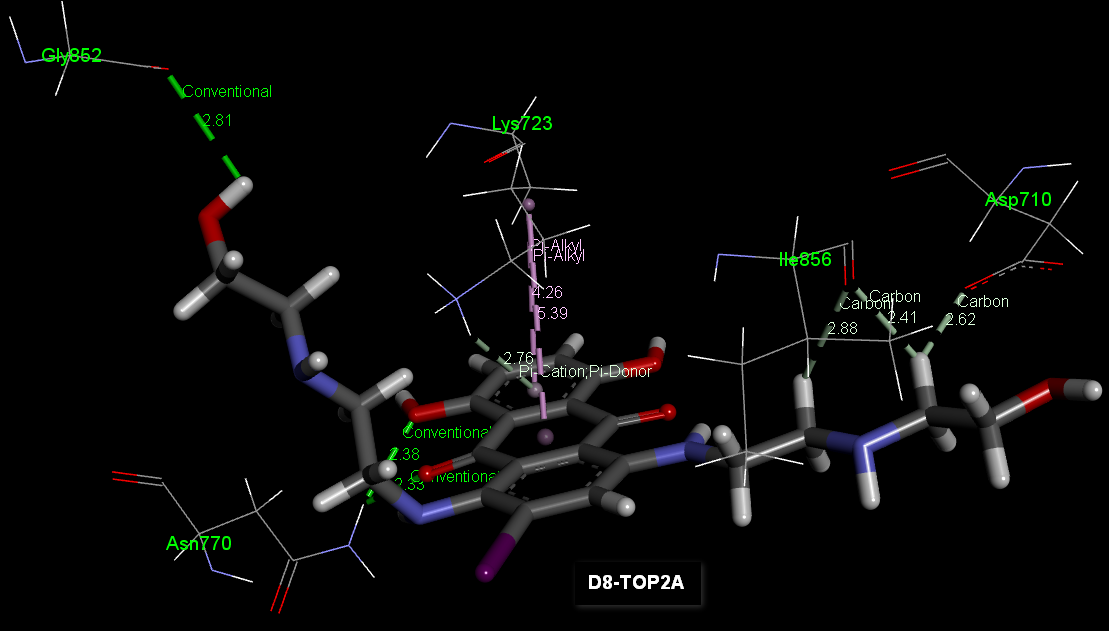


**Figure S13:** Non-bonded interactions in D8-TOP2A complex.
